# Experimental models of cerebral small vessel disease: Physiological constraints, translational challenges and future directions

**DOI:** 10.1113/JP290165

**Published:** 2026-06-05

**Authors:** Sophie Beaumont, Tanya Singh, Luana Campos Soares, Mootaz M. Salman

**Affiliations:** ^1^ Department of Physiology, Anatomy, and Genetics University of Oxford Oxford UK; ^2^ BHF Oxford Centre of Research Excellence University of Oxford Oxford UK; ^3^ Kavli Institute for NanoScience Discovery University of Oxford Oxford UK; ^4^ British Heart Foundation (BHF) ‐ UK Dementia Research Institute (UK DRI) Centre for Vascular Dementia Research University of Oxford Oxford UK

**Keywords:** AQP4, BBB, brain clearance, cerebral small vessel disease, glymphatic system, neurovascular unit, SVD

## Abstract

Cerebral small vessel disease (cSVD) is a chronic, progressive cerebrovascular disorder and the second most common cause of dementia after Alzheimer's disease. It accounts for approximately 20% of strokes, including a quarter of ischaemic strokes and nearly half of vascular dementias, representing a growing clinical and socio‐economic burden in ageing populations. Despite its prevalence, mechanistic understanding remains limited and disease‐modifying therapies are lacking. A major obstacle is the difficulty of interrogating disease progression *in vivo*, as the small calibre and deep location of affected vessels restrict assessment. Experimental modelling has therefore been central to advancing cSVD research. Rodent models have provided insight into vascular dysfunction, white matter injury and blood–brain barrier (BBB) impairment, but differ from humans in cerebrovascular anatomy, cellular composition and disease trajectory. Emerging *in vitro* approaches, including three‐dimensional cultures and microfluidic systems incorporating human vascular cells, offer improved experimental control and translational relevance, yet struggle to capture the slow progression of cSVD and its comorbidities such as hypertension and ageing. Most models therefore isolate pathological features rather than reproducing the integrated physiology of disease. In this review, we critically evaluate current *in vivo*, *in vitro* and *in silico* models of cSVD, highlighting their strengths and limitations. We identify the glymphatic system and brain clearance as underexplored but potentially unifying pathways linking vascular dysfunction, perivascular‐space enlargement and impaired fluid clearance. Incorporating glymphatic elements into advanced models may address key mechanistic gaps. Improving physiological fidelity in cSVD modelling will be essential for robust target identification and development of effective therapies.

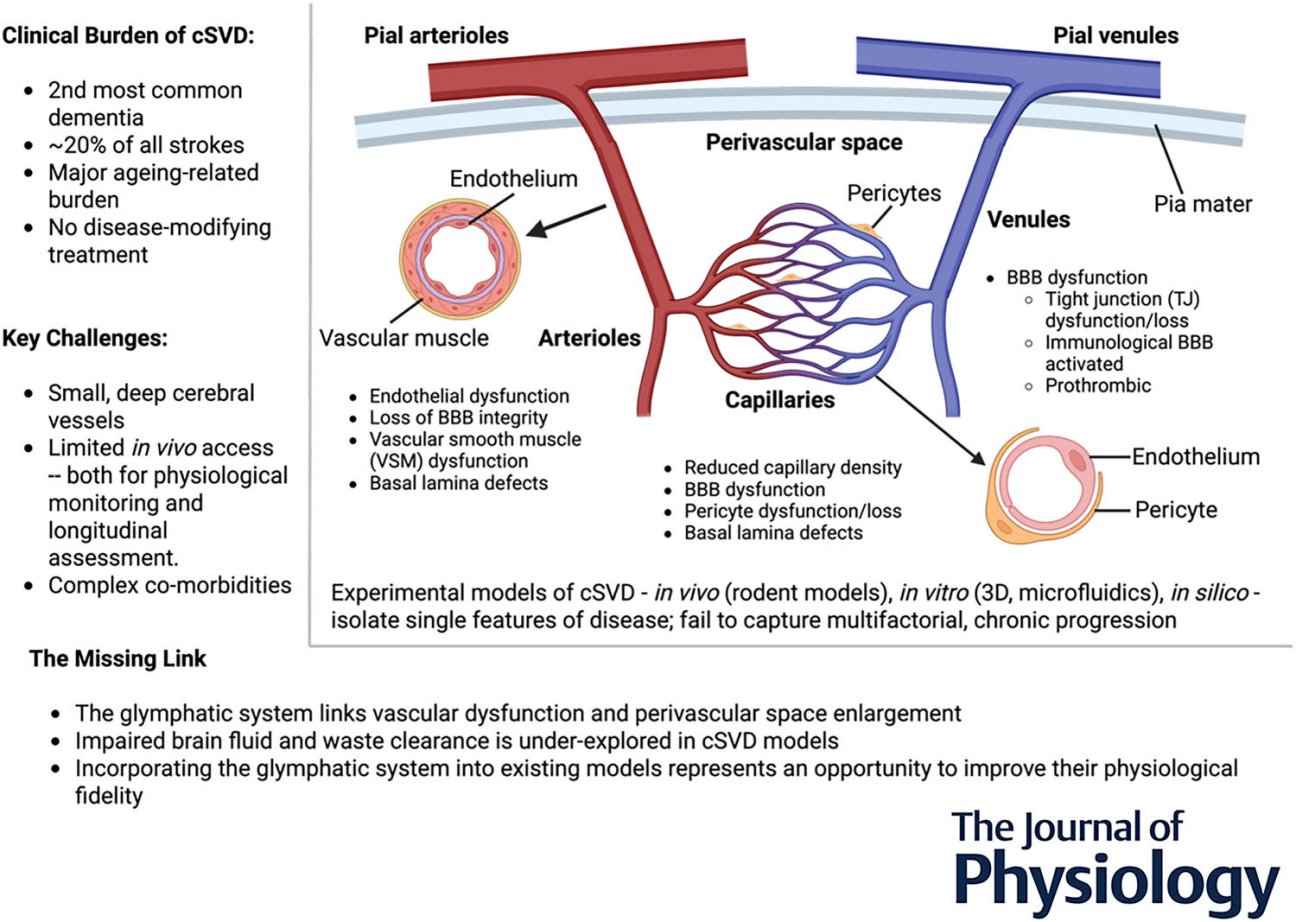

## Graphical abstract

**Abstract figure legend** Overview of cerebral small vessel disease (cSVD) pathophysiology and current modelling challenges. Left: clinical burden and key unresolved research questions. Right: neurovascular unit (NVU) architecture highlighting sites of cSVD pathology across arterioles, capillaries and venules, including interactions between endothelial cells, mural cells, astrocytes and neurons. The glymphatic system provides a mechanistic link between vascular dysfunction, perivascular space enlargement and impaired interstitial fluid clearance, representing an underexplored opportunity to enhance the physiological fidelity of cSVD models.

## Introduction

Cerebral small vessel disease (cSVD), also referred to as cerebral microangiopathy, is a heterogeneous group of chronic, progressive pathologies whose definition remains contentious due to complex underlying pathophysiological mechanisms. It is characterised by dysfunction of the brain's small penetrating arteries, arterioles, capillaries and venules, with consequent impairment of endothelial and blood–brain barrier (BBB) integrity that ultimately drives injury to both white and grey matter (Kremer et al., 2025). Pathological consequences are generally accepted to stem from secondary impacts on the brain parenchyma, rather than diseased blood vessels themselves (Wardlaw et al., 2013).

Despite being the second most common cause of dementia after Alzheimer's disease (AD), there are no disease‐modifying treatments that are currently available (Chojdak‐Łukasiewicz et al., 2021). In most cases, the condition is sporadic, and its aetiopathogenesis frequently involves comorbidities such as hypertension, AD and diabetes mellitus, though additional risk factors include sleep disruption, ageing, obesity, neuroinflammation and smoking (Wardlaw et al., 2021). The interplay between cSVD and its comorbidities is poorly studied and not fully understood.

Magnetic resonance imaging (MRI) is the standard diagnostic method, in which perivascular spaces around perforating vessels often become enlarged, fluid‐filled and visible on scans. However, this detection method can result in ‘invisible’ pathologies, such as microinfarcts, only seen microscopically, being overlooked. Post‐diagnosis, patients are advised to manage the condition by modulating known risk factors for cardiovascular disease, given the shared pathology with ischaemic stroke (Chojdak‐Łukasiewicz et al., 2021). As a major contributor to age‐related cognitive decline, cSVD is expected to impose an increasing socio‐economic burden as populations continue to age (Pantoni, 2010).

**Figure 1:**
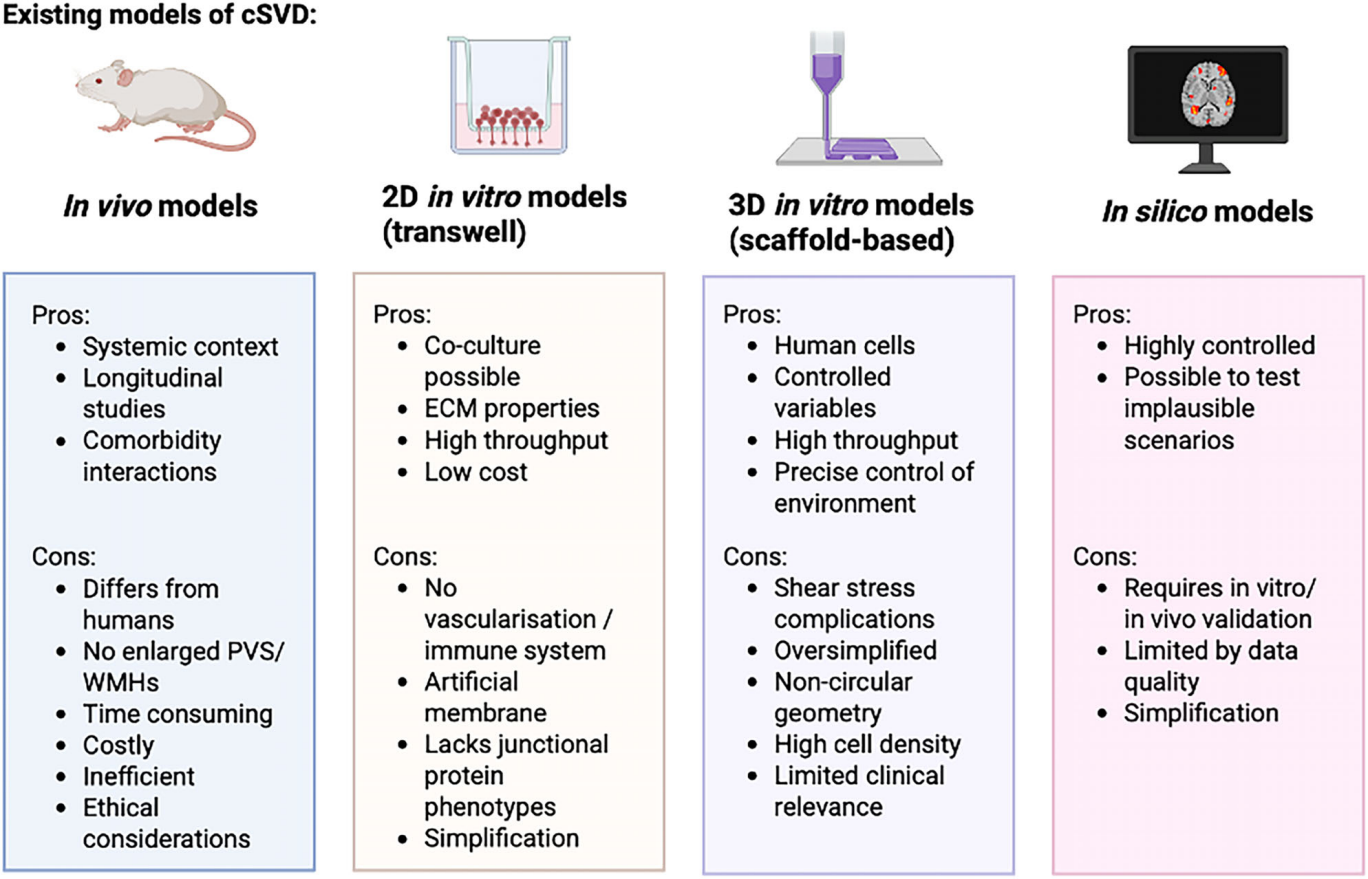
Overview of experimental approaches currently used to study cerebral small vessel disease (cSVD) The schematic illustration compares four major modelling strategies: *in vivo* animal models, two‐dimensional *in vitro* transwell systems, three‐dimensional scaffold‐based *in vitro* platforms, and *in silico* computational models. Each approach captures distinct aspects of cSVD pathophysiology while presenting specific limitations in physiological relevance, experimental control and translational applicability. PVS, perivascular spaces; WMHs, white matter hyperintensities.

Experimental modelling has been indispensable in advancing mechanistic understanding of cSVD. However, the intrinsic complexity of the disorder, characterised by slow progression, vascular heterogeneity and frequent systemic comorbidities, poses significant translational challenges. Much of our current knowledge derives from rodent models which, although highly informative, cannot fully recapitulate the cellular, vascular and molecular architecture of the human brain (Fig. 1).

Fundamental interspecies differences exist in cerebrovascular anatomy, cellular composition and gene expression profiles. Rodents exhibit distinct vascular branching patterns and microvascular organisation and differ in the relative abundance and spatial distribution of neural and glial cell populations. Importantly, transcriptomic analyses using single‐nucleus RNA sequencing (snRNA‐seq) have revealed quantifiable divergence in gene expression across homologous neural cell types between mouse and human cortex. Hodge et al. (2019) further demonstrated pronounced species‐specific differences in astrocyte morphology and transcriptional signatures, underscoring the limitations of direct extrapolation from rodent to human pathophysiology. In humans, not only is the olfactory artery absent in adults, but the anatomical origin of the posterior cerebral artery in the circle of Willis also differs from that of rodents, with important consequences for modelling stroke (Schröder et al., 2020). The cellular and molecular profile of brain vasculature has been investigated in both species (Vanlandewijck et al., 2018; Wälchli et al., 2024; Yang et al., 2022). Song et al. (2020) directly compared transcriptomics of microdissected brain vasculature of mouse and human. They found several species‐specific endothelial and pericyte‐enriched genes that are relevant for barrier transport and have consequences for drug delivery research. Astrocytes, which enwrap the vasculature with their endfeet, are demonstrably larger in humans than in mice (Cruz et al., 2026; Preman et al., 2021). Moreover, a recent study has compared the proteome of isolated endfeet between the two species finding that 75% of mouse receptors are also expressed in humans, as well as 54% of ligand–receptor pairs (Hill et al., 2025). These findings indicate the presence of murine‐specific perivascular proteins and raise the possibility that species‐specific components may also exist in humans.

Importantly, two hallmarks of cSVD, enlarged perivascular spaces and white matter hyperintensities, have not been robustly identified in rodent models (Stringer et al., 2021). The lack of appropriate experimental models has therefore been a major barrier to the identification of robust drug targets and the development of effective therapies for cSVD (Smith & Markus, 2020). In recent years, *in vitro* approaches to studying cSVD, including three‐dimensional culture systems and microfluidic platforms, have become increasingly accessible, alongside growing interest in computational modelling strategies (Fig. 1). Compared with traditional rodent models, these new techniques have several advantages that enable mechanistic insights at the level of cells and tissues. Alongside existing animal models, they have potential to improve efficiency of drug screening and translational accuracy of preclinical research due to lower costs and the inclusion of patient‐derived cells (Pierre et al., 2025). A parallel shift toward *in vitro* and human‐relevant modelling is also emerging at the regulatory level, with the U.S. Food and Drug Administration (FDA) recently signalling its intention to reduce reliance on animal testing in favour of advanced platforms such as organ‐on‐a‐chip systems and other new‐approach methodologies (Ingber, 2026). These developments reflect a broader recognition that complex human pathophysiology may require equally sophisticated experimental frameworks. Nevertheless, substantial challenges persist in modelling cSVD. A central difficulty lies in reproducing the slow, progressive and often subclinical evolution of the disease. The temporal dimension of cSVD, characterised by cumulative vascular stress, gradual endothelial dysfunction and evolving neurovascular uncoupling, is inherently difficult to capture in short‐term experimental systems. Moreover, the intertwined contributions of inflammation, endothelial dysfunction, extracellular matrix remodelling and vascular rarefaction are challenging to model simultaneously within reductionist platforms (Mustapha et al., 2019). In this review, we critically evaluate recent advances in experimental models of cSVD, outlining their respective strengths and limitations. We further examine the emerging role of brain clearance pathways and glymphatic function as an underrepresented yet potentially central component of cSVD pathophysiology. Integrating glymphatic physiology into experimental paradigms may provide new mechanistic insight into disease initiation and progression and help bridge persistent translational gaps in the field.

### Modelling cSVD

Models of cSVD frequently analyse an isolated aspect of the disease (e.g. hypertension). In doing so, they have limited applicability as the vast web of associated comorbidities is overlooked. Many models are also insufficiently humanised and cannot replicate aspects of human physiology including continuous unidirectional blood flow, protein expression patterns, extracellular matrix (ECM) mechanobiology and cylindrical geometries typical of blood vessels (Fig. 1).

### Rodent models

Rodent models remain central to experimental cSVD research and have provided valuable mechanistic insight through paradigms based on chronic hypoperfusion and ischaemic injury, hypertension‐driven vascular stress, or direct vascular damage (Mustapha et al., 2019).

Initial work using the spontaneously hypertensive rat–stroke prone (SHRSP) model revealed cSVD‐like features (Table 1), with evidence of cerebrovascular dysfunction characterised by increased vascular permeability and a predilection for haemorrhage and cortical softening at arterial boundary zones, particularly within the basal ganglia (Yamori et al., 1976). A later study suggested that endothelial cell (EC) dysfunction was intrinsic and independent of hypertension, challenging the traditional primacy of hypertension as the initiating driver of cSVD (Rajani et al., 2018).

**Table 1:** Representative animal models used to study cerebral small vessel disease (cSVD)

Model	Description	Pathological features	Considerations
Spontaneously hypertensive rat–stroke prone (SHRSP) (Collidge et al., 2004; Hannawi et al., 2021; Yamori et al., 2022)	Inbred; non‐surgical model of stroke Recapitulates lacunar stroke, cSVD and subcortical ischaemic stroke	Endothelial injuries/local BBB breakdown cause blood vessel damage Enlarged PVS, white matter damage and microinfarcts Haemorrhage in the context of malignant hypertension	Cardiac complications independent of cSVD Inbreeding; limited genetic diversity Normotensive cSVD not reflected Low physiological relevance: rapid disease progression Unknown molecular/genetic causes
ATP11BKO transgenic rat (Quick et al., 2022)	ATP11B phospholipase flippase protein homozygous knock‐out	Normotensive EC dysfunction Enlarged and distorted PVS, brain atrophy, and abnormal white matter Mutations and vessel damage	Global homozygosity, factors other than EC dysfunction may contribute to pathology Unknown pathological mechanism Low physiological relevance: mutation does not occur in humans
NOTCH3 transgenic mice (Baron‐Menguy et al., 2017; Huang et al., 2026; Joutel, 2025)	Mimics CADASIL, the most frequent cause of stroke and vascular dementia Number of cysteine residues in the extracellular domain are altered	Blood pressure‐independent reduction in lumen diameter and increased stiffness VSMC dysfunction No BBB leakiness or lacunar infarcts Reduced cerebral blood flow Mutations and vessel damage	Unknown pathological mechanism Low physiological relevance: recapitulates rare monogenic cSVD while many patient cases are sporadic
Fluorescent microsphere embolisation (Silasi et al., 2015)	Endovascular injection of fluorescent microspheres produces regional distribution of microocclusions as seen in cSVD Silasi et al. applied this technique in Thy1‐GFP mice; GFP expression in a neuronal subset enabled direct visualisation of microsphere‐induced changes to neuronal structure	Regional microocclusions recapitulating the distribution of small vessel occlusions seen in cSVD Disrupts neurons in white matter tracts, striatum and thalamus Vessel damage	Low physiological relevance: this endovascular approach produces an acute shower of microinfarcts not reflective of gradual accumulation of microocclusions in humans
Bilateral common carotid artery stenosis (BCAS) (Shibata et al., 2004; Wazny et al., 2025)	Reduction of blood flow from bilateral carotid arteries using microcoils	Cerebral hypoperfusion, white matter lesions, neuroinflammation and cognitive impairment White matter focused lesions	Better suited for white matter‐focussed and subcortical ischaemic vascular dementia studies
Bilateral common carotid artery occlusion (BCCAO) (Soria et al., 2013)	Ligation of bilateral common carotid causing occlusion	Cerebral hypoperfusion, white matter lesions, neuroinflammation and cognitive impairment More widespread damage	Represents ischaemic injury rather than progressive degeneration Hypoperfusion has been suggested not to be the trigger for cSVD

The table summarises commonly employed rodent models, grouped according to their primary pathogenic drivers, including hypoperfusion or ischaemic injury, hypertension, vascular damage, and genetic mutations affecting the vessel wall. While these models reproduce selected clinicopathological features of human cSVD, none captures the full complexity of the disease, and their relevance varies depending on the specific aspect of cSVD under investigation (Mustapha et al., 2019). BBB, blood–brain barrier; CADASIL, cerebral autosomal dominant arteriopathy with subcortical infarcts and leukoencephalopathy; EC, endothelial cell; GFP, green fluorescent protein; PVS, perivascular spaces; VSMC, vascular smooth muscle cell.

More recently, the ATP11B knockout (ATP11BKO) rat model has provided direct evidence for intrinsic EC dysfunction in cSVD, characterised by reduced endothelial nitric oxide synthase (eNOS) and Claudin‐5 expression, alongside upregulation of intercellular adhesion molecule 1 (ICAM‐1). Notably, this model recapitulates key pathological, neuroimaging and behavioural features of cSVD in a normotensive context, thereby challenging the traditional primacy of hypertension and positioning EC dysfunction as a primary pathogenic driver (Quick et al., 2022). While extrinsic stressors such as hypertension can precipitate or exacerbate endothelial dysfunction, they may not be strictly required for the development of cSVD‐like pathology. These findings strengthen the rationale for targeting endothelial integrity directly, rather than focusing exclusively on systemic vascular risk factor control. This has implications for diagnosis since studying diseased ECs in the absence of hypertension could help identify cSVD‐specific biomarkers.

A complementary body of research highlights the role of vascular smooth muscle cell (VSMC) dysfunction in cSVD pathogenesis. Cerebral autosomal dominant arteriopathy with subcortical infarcts and leukoencephalopathy (CADASIL) is the most common hereditary form of cSVD. It is caused by a series of characteristic mutations in the NOTCH3 receptor, which is expressed predominantly in mural cells of the brain vasculature, including VSMCs and pericytes, rather than ECs. These mutations typically result in a gain or loss of cysteine residues within the NOTCH3 extracellular domain (NOTCH3^ECD^), which disrupts its conserved disulphide bond pattern and promotes protein aggregation. This aligns with the observed accumulation of NOTCH3^ECD^ in CADASIL patients prior to clinical manifestation (Lee et al., 2023), suggesting that VSMC‐intrinsic signalling dysfunction constitutes a distinct pathogenic mechanism contributing to cSVD susceptibility. Consistent with this, recent conditional knock‐in models demonstrate that pericyte‐ and VSMC‐specific expression of the *Notch3* ^R170C^ variant independently recapitulates key CADASIL features, but through divergent pathways, with cell type‐specific vascular, neuroinflammatory and proteomic signatures (Huang et al., 2026).

Despite significant progress, no single animal model currently recapitulates the full spectrum of pathological, physiological and clinical features of the disease (Mustapha et al., 2019). Models commonly recapitulate a single aspect of cSVD, such as white matter damage or BBB dysfunction, though crossing existing strains may provide a more holistic representation. Existing models offer a system‐based representation of cSVD and combining strains could provide a clinically relevant route to unravel the interplay between associated comorbidities.

### *In vitro* models

There is a clear need for physiologically relevant *in vitro* models of cSVD. As outlined above, existing *in vivo* systems typically capture only discrete aspects of the pathology and do not fully recapitulate key features of human cerebrovascular physiology. In contrast, *in vitro* platforms that enable systematic and independent manipulation of disease‐relevant variables provide a powerful framework to interrogate causal mechanisms driving cSVD progression. An optimal model would incorporate physiologically relevant shear stress and extracellular matrix interactions, support unidirectional flow within a cylindrical microvascular geometry without artificial barriers, and be compatible with advanced imaging modalities and three‐dimensional tracking of labelled molecules (Salman et al., 2020).

Microfluidic modelling offers a promising approach to recreate vascular biology at the microscale, enabling precise manipulation of sub‐millimetre volumes under controlled conditions where physical and biological variables can be interrogated in isolation. Hydrogel‐based cell‐laden bioinks provide the mechanical and biomechanical cues required for 3D vascular growth and have recently been applied to model cerebral vasculature. ECs, thought to be key players in cSVD (Quick et al., 2022), and VSMCs were co‐cultured into a cylindrical vessel using this bioink (Gold et al., 2021). Sacrificial printing approaches overcome difficulties associated with *in vitro* 3D cell co‐culture (e.g. lack of small vessel structures). The model also has potential for humanisation. Introducing patient‐relevant cSVD mutations (e.g. *COL4A1/2*) into the endothelial component would allow disease‐relevant phenotyping in a geometrically realistic vascular environment (Table 2). However, the approach requires higher than relevant cell densities and may yield an asymmetric channel cross‐section impacting cell–cell interactions and ECM behaviour.

**Table 2:** Common genetic mutations associated with cerebral small vessel disease and their use in *in vitro* modelling

Gene	Wild‐type function	Type of mutations	Considerations for *in vitro* models
*COL4A1/2* (Ferguson et al., 2022)	Encodes α‐1 and α‐2 chains of type IV collagen	Missense are most common; glycine substitution in Gly‐X‐Y Familial rates range from 2% to 10%. Rare in sporadic cSVD: ∼1% 20% of *COL4A1* and 4% of *COL4A2* carriers show cSVD phenotype	Increased rigidity can change permeability of nutrients in the hydrogel Dominant negative effects Location of glycine substitution within the helix affects severity Possible compensatory mechanisms from α‐3 and α‐6 chains Difficult to model chronic, progressive pathology
*NOTCH3* (Yamashiro et al., 2025)	Guides the development of VSMCs and pericytes Maintains structural integrity of blood vessels, particularly small arteries	Cysteine mutation of EGF repeats in the extracellular domain (∼95%). Most commonly in EGF repeats 1–6 Disrupted disulfide bonds affect protein folding/stability Most common and best characterised monogenic form of cSVD Causes CADASIL	Long experiment times: weeks to months in cell culture Cell type restriction; primarily relevant for VSMCs and pericytes Mechanism unknown; toxic aggregation or loss of signalling or both
*HTRA1* (Ferguson et al., 2022; Yamashiro et al., 2025; Zhang et al., 2022)	Encodes a serine protease that regulates signalling pathways (e.g. TGF‐β, WNT and NOTCH) Involved in quality control, cell fate and regulation of angiogenesis	Dominant inheritance pattern for familial cSVD; heterozygous and homozygous mutations Homozygous *HTRA1* causes CARASIL Co‐existence with NOTCH3 9% of carriers show cSVD phenotype	HTRA1 may trigger secondary proteolytic events; complex to dissect Possible compensation from other HTRA‐family members HTRA1 is secreted; difficult to maintain stable local concentrations in hydrogels
*TREX1* (Richards et al., 2007; Wilms et al., 2022)	Affects TREX1 3–5 DNA exonuclease involved in clearing cytosolic nucleic acids	Causes RVCL‐S, a hereditary model for sporadic cSVD	Acute *in vitro* experiments may not capture chronic, low‐grade interferon signalling Causes systemic inflammation, *in vitro* models will lack circulating inflammatory factors Amongst the rarest monogenic cSVD causes; may lack relevance
*CTSA* (Guey et al., 2021; Yuan et al., 2024)	Impaired cathepsin cannot inactivate the bioactive peptide endothelin‐1 Endothelin‐1 builds up in cerebral vessels leading to atherosclerosis and damage to white matter	Heterozygous missense mutations; commonly R325C Causes CARASAL Extremely rare	*CTSA* mutations cause a multi‐system disease; cSVD is one of many manifestations not the primary phenotype Must be homozygous; more challenging to model Unclear vascular pathomechanism

The table compares the normal biological function of each gene, the types of disease‐associated mutations, and key considerations for incorporating these variants into experimental models of cSVD. CADASIL, cerebral autosomal dominant arteriopathy with subcortical infarcts and leukoencephalopathy; CARASAL, cathepsin A‐related arteriopathy with strokes and leukoencephalopathy; CARASIL, cerebral autosomal recessive arteriopathy with subcortical infarcts and leukoencephalopathy; EGF, epidermal growth factor; HTRA, high‐temperature requirement A; RVCL‐S, retinal vasculopathy with cerebral leukoencephalopathy and systemic manifestations; VSMC, vascular smooth muscle cell.

*In vitro* models offer a complementary approach to studying cSVD mechanisms. The capacity to manipulate individual variables while holding others constant allows independent factors driving cSVD to be examined in a controlled manner and helps pinpoint mechanisms that contribute to disease progression. This level of causal interrogation is difficult to achieve in animal models, where multiple physiological processes are inherently intertwined. The increased physiological complexity of advanced *in vitro* models relative to traditional cell culture techniques should allow for improved target validation and reduced development of drugs with off‐target effects (McCloskey et al., 2024). Further, monogenic cSVD shows variable penetrance and expressivity (Ferguson et al., 2022); not all carriers go on to develop the disease and those that do show varied clinical severity. Analysis of modifiable risk factors may help determine how environmental and lifestyle factors interact with underlying genetic mutations to drive disease. *In vitro* models allow for risk factors such as diabetes, hypertension, smoking and inflammation to be considered. In the case of diabetes, for example, observing the effect of increased glucose in cell culture media beyond the normal 5.5 mM could be used to model the downstream molecular consequences of hyperglycaemia on vascular and glial function.

A range of induced pluripotent stem cell (iPSC)‐derived co‐culture systems have been developed to model the cellular complexity of the BBB *in vitro*, with increasing levels of biological fidelity. At the structural level, co‐culture of brain microvascular endothelial cells (BMECs), pericytes and astrocytes embedded in a Matrigel‐based 3D ECM can recapitulate the anatomical and physiological properties of the human BBB, approximating the cellular composition of the neurovascular unit (NVU) (Ferguson et al., 2022). A complementary transwell‐based model uses a collagen hydrogel containing encapsulated astrocytes layered beneath iPSC‐derived endothelium (Pinals & Tsai, 2022). The increased concentration of collagen confers rigidity and resistance to contraction as well as cell selection for greater adherence, which prevents detachment during use. Beyond structural recapitulation, these models can also capture dynamic BBB functions. The µSiM tissue chip platform, a microphysiological system enabled by an ultrathin silicon nanomembrane, has been used to demonstrate that pericyte inclusion influences leukocyte transmigration across the BBB under inflammatory conditions such as sepsis (Pinals & Tsai, 2022). Full reconstruction of NVU architecture and signalling dynamics nonetheless remains an outstanding challenge across all these platforms.

A key advantage of iPSC technology is its ability to incorporate patient‐specific genetic variation, enabling disease modelling beyond generic cellular co‐culture. For example, iPSCs derived from individuals carrying *COL4A1/2* mutations have been differentiated into brain endothelial‐like and mural cells and co‐cultured in transwell systems, recapitulating disease‐relevant phenotypes, including extracellular matrix abnormalities (Domocos et al., 2026; Marazzi et al., 2025). Pharmacological inhibition of matrix metalloproteases (MMPs) partially restored endothelial function, identifying MMP activity as a potential therapeutic target and establishing the platform as a viable tool for both mechanistic interrogation and high‐throughput drug screening (Al‐Thani et al., 2023).

The composition of the ECM is a key determinant of how accurately *in vitro* models reproduce native vascular structure and function; it should be selected with the specific mutation under study in mind. For *COL4A1/2* mutations, incorporating collagen IV into hydrogel matrices is particularly important as this is the primary affected protein. Options range from Matrigel, pure collagen IV hydrogels or custom bio‐printed inks with ECM proteins in defined ratios. For *HTRA1* deficiency, matrix composition can be tailored to include relevant proteolytic substrates, such as fibronectin and decorin, to capture downstream effects on perivascular ECM remodelling. The ability to systematically vary ECM composition and directly compare mutant and isogenic wild‐type conditions provides a level of experimental control that is difficult to achieve *in vivo* and represents a key advantage of these emerging platforms (Table 3).

**Table 3:** Comparison of experimental models used to study cSVD

	Advantages	Disadvantages	Translational relevance
*In vivo* models: animals (Baron‐Menguy et al., 2017; Lee et al., 2023; Mustapha et al., 2019; Quick et al., 2022; Smith & Markus, 2020)	Provides whole‐system context; enables interactions between the glymphatic system, vascular network and immune system Ability to examine interactions of cSVD with its comorbidities (e.g. obesity, diabetes mellitus and Alzheimer's) Real‐time imaging (e.g. two‐photon microscopy and MRI) provide visualisation in live rodents Longitudinal studies allow study of chronic progression and impact of therapeutic intervention over time	No single model can represent cSVD fully Challenging experiments to perform results in possible exclusion of data and minimal reproducibility Breeding of transgenic mice may reduce group variability by controlling external factors (e.g. exercise and diet) which will be affected in human studies Key differences between humans and rodents (e.g. lifespan, brain size, vessel dimensions and grey–white matter ratio)	Microvascular structural alterations White matter injury Altered cerebral blood flow regulation Longitudinal MRI and two‐photon microscopy allow non‐invasive tracking of white matter hyperintensities and cerebral blood flow over timeRecent work has developed a dedicated translational mouse model of BBB leakage in cSVD driven by metabolic dysfunction, demonstrating that diet‐induced metabolic stress recapitulates clinically relevant patterns of barrier breakdown (Jia et al., 2025)
*In vitro* models: microfluidics (Campisi et al., 2018; Gold et al., 2021; McCloskey et al., 2024; Soria et al., 2013)	Controlled environment; allows precise manipulation of variables (e.g. flow dynamics, pressure, cellular components) Increased translational relevance; incorporation of hiPSC‐derived endothelial cells, astrocytes and mural cells High throughput testing; useful for screening drugs and testing interventions Tractable, simple and flexible Small volumes allow for fine control over distances, tissue organisation and mechanical cues Lower risk of contamination, consumption of reagents and throughput efficiency compared with cell culture	Cultured cells are susceptible to shear stresses. Higher flow rates can cause cell detachment/death. Slower flow rates do not represent the dynamic system. Flow rates should accurately represent arterial pulsation and appropriate rates Limited ability to replicate progressive nature of cSVD Replicated blood microvessels may not be sufficient to study macro‐sized vessels (e.g. arteries and aorta) Cross‐sectional channel geometry may not be circular Cannot replicate pulsatile haemodynamics of *in vivo* cerebral blood flow Shear stress profiles do not reflect the magnitude or temporal dynamics of physiological cerebrovascular conditions Oversimplification of disease; removed from the physiological context	BBB dysfunction Microvascular structural alterations Interstitial fluid flow Enable measurement of features difficult to isolate *in vivo*; for example, barrier permeability, tight junction integrity and endothelial responses to flow
*In vitro* models: transwell systems (Pinals & Tsai, 2022)	Allows cell co‐culture and representation of extracellular matrix properties Screening platform for drug testing	Lacks simulation of haemodynamic forces created by blood flow Cannot recapitulate phenotypes resulting from junctional proteins and transporters Contains artificial membranes	BBB dysfunction (particularly paracellular permeability and tight junction protein expression) Best used prior to, or in parallel with, microfluidic validation
*In silico* models (Cuadrado‐Godia et al., 2018; Shityakov & Förster, 2018; Šutalo et al., 2015)	Can be customised to test and measure many scenarios including those not possible with *in vivo*/vitro models Various simulated conditions possible allowing for improved ability for mechanistic insights A single variable could be changed at a time; highly controlled	The system modelled is highly complex (e.g. non‐linearity of the Navier–Stokes equation used to model blood flow dynamics/tissue perfusion) Models must be simplified and struggle to capture biological complexity Commonly built on empirical patient data obtained from small sample sizes Must eventually be validated with microfluidic/rodent models	Altered cerebral blood flow regulation (e.g. how changes in small vessel geometry, wall stiffness or branching architecture affect perfusion distribution) Useful in generating hypotheses to be tested *in vivo* or *in vitro*

The table summarises the main advantages and limitations of *in vivo* animal models, *in vitro* platforms (including microfluidic and transwell systems) and *in silico* approaches. Translational relevance is assessed across four key pathological dimensions of cSVD: microvascular structural alterations, white matter injury, BBB dysfunction and altered cerebral blood flow regulation. Owing to their complementary strengths, a combination of these models is required to dissect the genetic, molecular and cellular mechanisms underlying cSVD and to support the development and validation of preventive and therapeutic strategies. BBB, blood–brain barrier; hiPSC, human‐induced pluripotent stem cell.

Despite these advances, conventional transwell systems lack key physiological parameters, particularly shear stress and haemodynamic forces, and often fail to fully reproduce *in vivo* endothelial characteristics such as robust junctional protein expression (e.g. occludin) and transporter profiles (e.g. GLUT‐1). To address these limitations, microfluidic ‘brain microvessel‐on‐a‐chip’ systems have been developed (Salman et al., 2020). These 3D platforms generate a perfusable endothelial‐lined lumen within an ECM scaffold, allowing controlled unidirectional flow and real‐time 3D imaging through reagent access from the surrounding matrix. Integrating patient‐derived iPSC endothelial and mural cells into such microfluidic architectures would represent a significant advance, enabling more physiologically relevant modelling of cSVD microvascular pathology under flow conditions and facilitating high‐resolution structural and functional analyses.

Beyond model architecture, the genetic accuracy of cells is equally critical to faithful cSVD recapitulation. By controlling genetic background and allowing direct comparison of mutant and corrected lines, CRISPR/Cas9‐based genome editing combined with isogenic controls provides a rigorous framework for analysing the causal effects of cSVD‐associated variants, particularly in iPSC‐derived ECs and pericytes relevant to BBB function (Hsu et al., 2014; Wardlaw et al., 2013). This enables causal genetic inference by introducing or correcting specific disease‐associated variants (e.g. *NOTCH3*, *HTRA1*, *COL4A1/2*). Isogenic controls eliminate background genetic variability, increasing sensitivity to detect subtle vascular or BBB phenotypes. Although off‐target editing, clonal selection artefacts, and the limited capacity of iPSC models to replicate ageing and the polygenic, environmental factors prevalent in sporadic cSVD limit this technique (Kilpinen et al., 2017), it increases sensitivity for minor molecular and cellular abnormalities. Translational significance therefore depends on ethical and scientific rigour, including careful validation and suitable model selection.

Endothelial dysfunction, oxidative stress and impaired neurovascular unit signalling are all caused by ageing, which is the single strongest risk factor for cSVD (Pantoni, 2010; Wardlaw et al., 2019). Unfortunately, these long‐term systematic processes are challenging to replicate with *in vitro* models since they typically rely on young or immortalised cells. Although they simplify vascular architecture and chronic disease progression, *in vitro* BBB models (such as transwell, microfluidic and 3D co‐culture systems) are useful for studying tight junction disruption, pericyte loss and increased membrane permeability (Montagne et al., 2015). Collectively, these *in vitro* approaches are ethically consistent with the 3 Rs principle, minimising live animal use while incorporating human‐relevant biology. However, they necessitate rigorous donor consent, data protection and transparent acknowledgement of translational limits, particularly when employing iPSC‐derived or organoid‐based systems.

Complementing these experimental approaches, *in silico* modelling is becoming a powerful tool to understand neurological pathology and has potential to enhance *in vitro* models (e.g. molecular dynamics simulations of BBB pathology (Campisi et al., 2018) or fractal geometry models for cerebral blood flow (Shityakov & Förster, 2018)). However, accurately simulating cSVD using vascular flow modelling still presents several unique challenges, including highly complex vessel geometry, rapid fluid–solid interactions, blood vessel pulsatility and the non‐Newtonian nature of blood. Computational approaches are considered most useful in biomarker identification since reliable diagnostic methods are lacking due to the disease's complex manifestation. Emerging diagnostic tools commonly assess disease severity using machine learning approaches applied to image classification (Cuadrado‐Godia et al., 2018; Hu et al., 2024; Šutalo et al., 2015).

*In vitro* models offer precise, causal interrogation of specific cell types, genetic variants and microenvironmental conditions typically obscured by biological complexity *in vivo*. However, many aspects of cSVD pathophysiology are inherently systemic and multicellular. Processes such as vascular remodelling, chronic haemodynamic stress, neurovascular uncoupling and the progressive accumulation of white matter injury unfold over months. They involve coordinated interactions between the vasculature, immune system and brain parenchyma that cannot currently be reconstituted *in vitro*. *In vivo* models therefore remain essential for longitudinal studies of disease progression and whole‐organism therapeutic evaluation. The translational relevance of each platform also differs systematically across key pathological dimensions of cSVD. Evaluation of cSVD models should consider pathological and neuroimaging resemblance to human disease, including recapitulation of white matter hyperintensities, enlarged perivascular spaces and microinfarcts visible on MRI. Models should also support therapeutic testing, enabling longitudinal intervention, pharmacological manipulation of disease‐relevant targets and quantification of functional outcomes (e.g. cognitive deficits). Translational fidelity would also be enhanced by incorporation of disease‐relevant comorbidities (including ageing, hypertension, diabetes mellitus and chronic neuroinflammation) since these systemic factors shape cSVD progression in humans but are frequently absent in experimental systems. No single platform currently addresses all dimensions (Table 3). Robust mechanistic conclusions in cSVD research will therefore require convergent evidence across complementary experimental systems.

### Interactions at the capillary level

cSVD is fundamentally a disorder of the microvasculature, yet most experimental models remain reductionist, focusing on isolated cell types or discrete pathological features. The small arterioles and capillaries affected in cSVD constitute the anatomical and functional core of the NVU, an integrated system comprising ECs, VSMCs, pericytes, astrocytes and neurons that collectively regulate cerebral blood flow, BBB integrity and interstitial fluid dynamics. cSVD pathology is therefore unlikely to arise from dysfunction within any single cellular compartment but instead reflects disruption of coordinated signalling across this multicellular network. Dissecting these cell type‐specific and, critically, intercellular mechanisms is essential for the development of models that faithfully capture disease biology and enable meaningful translational insight.

Capturing this multicellular complexity experimentally remains a major challenge. Most *in vivo* rodent models recapitulate only a subset of pathological features, rather than the integrated NVU dysfunction characteristic of human cSVD. Recent work has begun to address this limitation. Tian et al. (2025) developed a mouse model of focal ischaemia‐induced vascular dementia in which intracranial delivery of the vasoconstrictor *N* ^5^‐(1‐iminoethyl)‐l‐ornithine (L‐NIO) generates multiple small cortical infarcts. Using single‐nucleus RNA sequencing across ECs, pericytes, VSMCs, astrocytes, microglia and neurons, they reconstructed the NVU interactome at single‐cell resolution. Their findings demonstrate that vascular pathology cannot be attributed to a single cellular compartment, but instead emerges from the disruption of coordinated multicellular signalling across the NVU.

Pericyte loss and dysfunction are increasingly recognised as critical components of cSVD progression. Beyond their structural role in microvascular stability and basement membrane remodelling, pericytes are active regulators of capillary diameter. *In vivo* two‐photon imaging has demonstrated that pericyte‐bearing capillaries dilate before penetrating arterioles following sensory input (Hall et al., 2014). In ischaemia, this contractile function becomes pathological: pericytes first constrict capillaries and subsequently die in rigor, contributing to persistent reduced blood flow and BBB damage (Hall et al., 2014). In the context of cSVD, these findings position pericyte dysfunction as a mechanistic target. Quantitative single‐vessel analysis of human cortical microvasculature across increasing cSVD severity has demonstrated that pericyte loss, assessed by platelet‐derived growth factor receptor β (PDGFRβ) signal, precedes endothelial activation across increasing disease severity (Chagnot A et al., 2026). The study showed near‐complete pericyte depletion observed in severely affected cases before vascular adhesion molecule upregulation becomes evident. This may indicate an early window where pericyte function could be restored before more widespread vascular damage becomes established. At the molecular level, pericytes interact directly with ECs to regulate BBB integrity and modulate perivascular fluid dynamics through aquaporin‐4 (AQP4) (Cao et al., 2025).

*In vitro*, multicellular co‐culture systems incorporating iPSC‐derived ECs, pericytes and astrocytes have demonstrated that BBB integrity is an emergent property of coordinated signalling across multiple cell types, rather than an intrinsic feature of the endothelium alone (Ferguson et al., 2022). Inclusion of pericytes in microphysiological BBB models, for example, modulates endothelial responses under inflammatory conditions, including leukocyte transmigration, in ways that cannot be predicted from endothelial monocultures (McCloskey et al., 2024). Microfluidic organ‐on‐a‐chip platforms further extend this approach by enabling interrogation of shear stress‐dependent endothelial–pericyte crosstalk under conditions that more closely approximate the *in vivo* capillary environment, including controlled unidirectional flow within cylindrical geometries (Salman et al., 2020). Moving forward, experimental platforms should aim to reconstruct the NVU as an integrated system, capturing pericyte–endothelial, astrocyte–endothelial and broader neurovascular interfaces central to cSVD pathophysiology. Coupling such systems with single‐cell transcriptomic resolution will be critical to resolve cell type‐specific contributions within this multicellular network.

### The glymphatic system as a candidate pathway linking cSVD to comorbid disease

The continued lack of effective therapies for cSVD highlights the limitations of existing experimental models. Consequently, robust pharmacological targets and scalable high‐throughput screening platforms remain scarce, making systematic lead identification challenging. This situation is further compounded by an incomplete mechanistic understanding of cSVD, which restricts the translation of experimental findings into therapeutic strategies.

**Figure 2:**
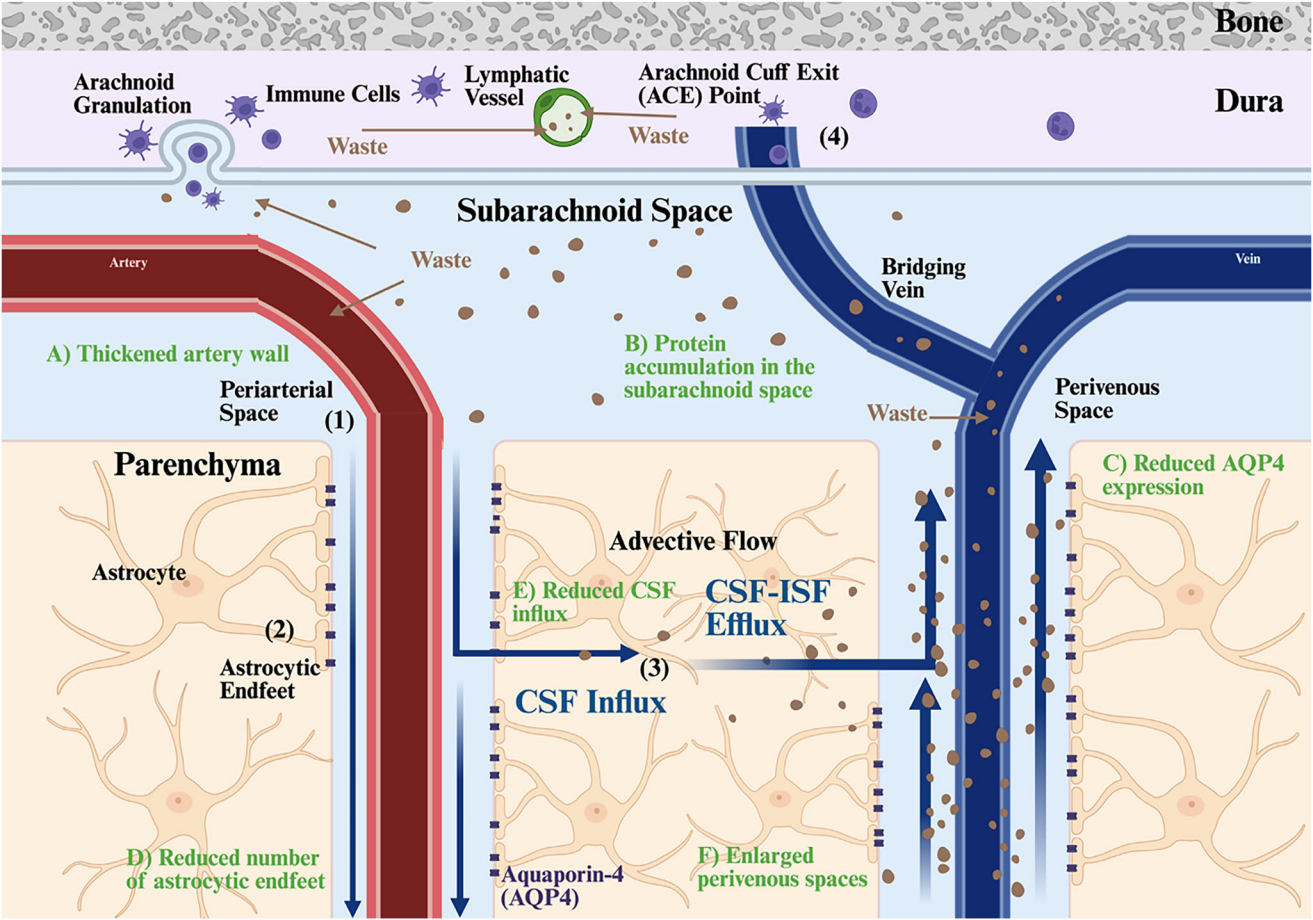
Graphical depiction of the glymphatic system and proposed impairment of glymphatic transport during cerebral small vessel disease (cSVD) (1) Cerebrospinal fluid (CSF) flows through perivascular spaces (PVS), created by vascular endfeet of astrocytes, with less resistance than in the neuropil (Boster et al., 2024; Simard et al., 2003). (2) Astrocytic endfeet surround the cerebral vascular bed to create a transport network that moves (CSF) from the subarachnoid space along arterial vasculature, deep into the interstitium. The water channel, AQP4, has densely localised expression on astrocytes at the PVS–parenchyma interface and facilitates CSF entry to the interstitium where it mixes with interstitial fluid (ISF) (Nagelhus & Ottersen, 2013). (3) Propelled by cardiac rhythm‐linked pulsations of the artery wall, CSF containing interstitial waste products moves to PVS through the parenchyma (Mestre et al., 2018). (4) Fluid containing glymphatic waste drains to dural lymphatic vessels via arachnoid cuff exit (ACE) points, which facilitate communication between the dura and parenchyma. Some fluid also directly drains from the subarachnoid space to the dura via arachnoid granulations surrounded by localised immune cells for surveillance. The CSF–ISF fluid is finally exported to extracranial lymphatics (Aspelund et al., 2015; Mortensen et al., 2019). Six key pathological changes that impair interstitial fluid dynamics and waste clearance from the brain parenchyma are shown in green (Mortensen et al., 2019; Tang et al., 2022). (A) Thickened artery wall. In cSVD, progressive arteriopathy results in thickening and stiffening of arterial walls. (B) Protein accumulation in the subarachnoid space. Impaired clearance mechanisms observed in cSVD result in protein accumulation which may physically obstruct flow and impair glymphatic function. (C) Reduced aquaporin‐4 (AQP4) expression. In cSVD, AQP4 expression and polarisation are frequently disrupted which reduces the efficiency of CSF‐ISF exchange. (D) Reduced number of astrocytic endfeet. Reduced coverage of cerebral vessels by astrocytic endfeet can result from chronic inflammation, membrane abnormalities or astrocyte injury. This results in reduced surface area available for AQP4‐mediated fluid transport. (E) Reduced CSF influx. Combined arterial wall thickening and protein accumulation with reduced vascular pulsatility, astrocytic endfeet and AQP4 expression impairs entry of CSF into periarterial spaces. (F) Enlarged perivenous spaces. These are a radiological hallmark of cSVD and may represent chronic obstruction of drainage pathways, increased vascular permeability with fluid accumulation or compensatory dilation in response to impaired clearance. Created with BioRender.com.

Glymphatic dysfunction has been reported in association with several neurological disorders, most notably AD and Parkinson's disease (Buccellato et al., 2022). This concept arises from a fundamental feature of the CNS: the absence of conventional lymphatic vessels within the brain parenchyma. Instead, the brain relies on specialised fluid transport pathways, operating alongside the BBB, which tightly regulates molecular exchange to protect neural tissue from pathogens and systemic metabolic fluctuations (Rasmussen et al., 2022). The BBB forms a series of tight junctions to prevent fluid exchange between the CNS and the bloodstream/peripheral extracellular fluid (Thomas, 2019). In the absence of fluid influx, diffusion processes were thought to be responsible for fluid distribution within the CNS (Abbott, 2004; Abbott, 2005; Syková & Nicholson, 2008). The glymphatic hypothesis, first proposed in 2012 after the availability of dynamic contrast‐enhanced MRI (DCE‐MRI) (Iliff et al., 2013; Iliff et al., 2012) offers an alternative explanation based on advection (Fig. 2). In early foundational studies, DCE‐MRI enabled direct visualisation and functional tracking of glymphatic transport across the rodent brain by following the movement of gadolinium‐based contrast agents administered into the cerebrospinal fluid. Critically, the study implicated AQP4 in this process and demonstrated paravascular transport of amyloid‐β, establishing the first proposed link between glymphatic drainage and Alzheimer's disease (Iliff et al., 2012). Since its initial description, the glymphatic hypothesis has generated considerable debate. In particular, the extent to which AQP4 directly mediates cerebrospinal fluid transport and whether glymphatic activity is substantially enhanced during sleep remain areas of active discussion and ongoing investigation (Abbott et al., 2018; Iliff et al., 2013; Markou et al., 2022; Smith et al., 2017). Additionally, the relative contribution of intramural periarterial drainage (IPAD) to brain waste clearance remains unclear. IPAD involves the elimination of interstitial fluid and soluble metabolites along basement membranes in the walls of capillaries and arteries, potentially driven by vasomotion rather than arterial pulsations, and may represent a complementary or alternative pathway to the glymphatic system (Aldea et al., 2019; Carare et al., 2020).

In recent years, a hypothesis of ‘CNS interstitial fluidopathy’ has emerged to describe disorders that exhibit impaired interstitial fluid dynamics (Singh et al., 2023; Taoka & Naganawa, 2021). The hypothesis suggests that many neurodegenerative and cerebrovascular diseases share an underlying mechanism: dysfunction of the brain's fluid clearance systems. A mechanistic link between the glymphatic system and cSVD remains undetermined, though growing evidence suggests glymphatic dysfunction is directly related to disease progression. Using the diffusion tensor image analysis along the perivascular space (DTI‐ALPS) index as a non‐invasive marker of glymphatic function, Tang et al. (2022) demonstrated that reduced glymphatic activity is independently associated with executive, attention and memory decline in cSVD patients, though not with language or visuospatial function, representing the first direct analysis of this relationship. It highlights the ALPS index as a potentially valuable predictive tool for cognitive decline in cSVD and supports the incorporation of glymphatic elements into experimental models. For example, *COL4A1/2* may compromise basement membrane permeability, directly affecting fluid transit across vessel walls. *HTRA1* deficiency may lead to ECM protein accumulation in perivascular spaces, obstructing drainage pathways. These gene‐specific mechanisms converge on a common pathophysiological endpoint: disrupted interstitial fluid dynamics. Indeed, several physiological changes are shared between cSVD and glymphatic disturbance, including increased visibility of perivascular spaces and progressive deterioration of the cerebrovascular bed (Singh et al., 2023; Taoka & Naganawa, 2021). In addition, established comorbidities of cSVD progression coincide with glymphatic dysfunction, such as hypertension. It has been demonstrated that glymphatic clearance is impaired in spontaneously hypertensive rats (SHR), highlighting the potential importance of the glymphatic system in cSVD development (Mortensen et al., 2019).

Despite the possible link, understanding of the glymphatic system is limited by experimental inaccessibility (Bohr et al., 2022; Taoka et al., 2017). In 2017, the DTI‐ALPS index was the first alternative proposed to invasive gadolinium‐enhanced MRI from original studies (Taoka et al., 2017). Despite the advancement, both methods are observational and lack the experimental control possible with *in vitro* models. There is, therefore, an urgent need for *in vitro* cSVD models that incorporate glymphatic features. Refining cSVD models by introducing aspects of glymphatic (dys)function, namely interstitial fluid flow, would allow a previously understudied but possibly central aspect of disease progression to be investigated. *In vitro* cSVD modelling is also useful for therapeutic screening and could be used to investigate glymphatic targets with a suspected role in cSVD, such as AQP4 (Peng et al., 2023). In this case, activators and/or inhibitors of the glymphatic system could be tested on a background model of cSVD to determine how changes in glymphatic flow affect disease severity. For this approach to progress, however, validation of targets with potential to modulate glymphatic function remains necessary (Box 1).

**AQP4 as a therapeutic target for cSVD**

The progressive nature of cSVD provides a comparatively broad therapeutic window, creating an opportunity to delay disease onset or attenuate progression. Increasing evidence linking cSVD to impaired brain clearance and glymphatic dysfunction has brought AQP4, the astroglial water channel central to perivascular fluid exchange, into focus as a mechanistically grounded and potentially tractable therapeutic target (Lee et al., 2024; Peng et al., 2023). Brain clearance and glymphatic dysfunction may act both as a driver of cSVD progression and as a predisposing factor for disease initiation, raising the possibility that restoration of perivascular fluid dynamics through modulation of AQP4 could modify the disease trajectory.

Supporting this concept, fibrinogen depletion in a transgenic cerebral amyloid angiopathy mouse model restored polarized AQP4 localisation and reduced amyloid burden (Singh et al., 2025), suggesting that targeting upstream mediators of AQP4 depolarisation may represent an additional and potentially more tractable therapeutic strategy. Together, these findings position AQP4 not only as a biomarker of glymphatic disruption but also as a mechanistically grounded candidate for disease‐modifying intervention in cSVD (Singh et al., 2025).

Developing pharmacological modulators of AQP4 remains a considerable challenge. Therapeutic delivery across the BBB is intrinsically constrained, and candidate compounds frequently exhibit limited specificity and off‐target activity. Recently, TGN‐073 has been proposed as a potential therapeutic agent to enhance glymphatic function, based on encouraging findings in a rat model (Alghanimy et al., 2023). However, a recent study examining AER‐270 and TGN‐020, previously assumed to be selective AQP4 inhibitors, has fundamentally challenged this view. Although these compounds were originally identified through *in silico* screening and validated using *Xenopus laevis* oocyte assays, Unger et al. (2024) demonstrated across multiple orthogonal systems that neither directly inhibits AQP4 in recombinant proteoliposome assays or mammalian cell models. Binding studies further revealed that AER‐270 exhibits markedly lower affinity for AQP4 than expected from reported IC_50_ values, suggesting that prior *in vivo* effects likely reflect off‐target mechanisms. These findings indicate that AER‐270 and TGN‐020 should not be used to infer AQP4‐dependent effects in the brain; by extension, similar caution may be warranted for TGN‐073. More broadly, this work underscores the necessity of rigorously validated *in vitro* platforms to accurately interrogate glymphatic mechanisms and confirm target engagement before therapeutic translation (Unger et al., 2024). This issue is particularly important given that no aquaporin‐targeting drugs are currently approved for human use, yet a prodrug of AER‐270 has entered phase I clinical trials for the prevention of brain oedema following stroke (Kitchen et al., 2020; Sun et al., 2022). The identification of compounds that selectively and directly modulate AQP4 would provide essential tools to interrogate glymphatic function in the context of cSVD and accelerate the rational development of therapies for cSVD and related disorders of CNS interstitial fluid homeostasis (fluidopathies).

### Conclusions and future directions

Our current understanding of cSVD remains fragmented and is shaped largely by experimental models that capture only isolated features of this complex and slowly progressive disorder. The limited physiological fidelity of many existing systems has constrained identification of causal mechanisms and likely contributed to the absence of effective disease‐modifying therapies. Translational interpretation is further complicated by fundamental differences between experimental systems and human cerebrovascular physiology. Rodent models diverge in white matter composition, vascular architecture and endothelial gene expression, whereas *in vitro* platforms lack systemic context, haemodynamic pulsatility and disease‐relevant cellular ageing. Notably, the two principal radiological hallmarks of human cSVD, enlarged perivascular spaces and white matter hyperintensities, remain inadequately reproduced in preclinical models. These limitations emphasise the need for convergent evidence across complementary platforms, rather than reliance on any single system. Within this framework, rigorously designed *in vitro* models are particularly valuable, as they enable precise control of disease‐relevant variables and direct interrogation of human cell‐specific mechanisms not accessible *in vivo*.

Recent advances in multicellular culture systems, organ‐on‐chip platforms and computational modelling provide a realistic opportunity to move beyond descriptive pathology towards mechanism‐driven target identification and more efficient therapeutic evaluation. Human‐relevant systems, including patient‐specific iPSC‐derived neurovascular models, combined with multi‐omics approaches integrating transcriptomic, proteomic and epigenetic readouts, offer a level of mechanistic resolution not previously achievable. Parallel advances in high‐resolution vascular imaging, including 7T MRI and two‐photon microscopy, will be essential to validate experimental findings and bridge the gap between model systems and human disease.

Incorporating principles of brain clearance and glymphatic physiology into experimental paradigms represents a particularly compelling next step. These systems sit at the intersection of vascular integrity, astrocyte function, interstitial fluid dynamics and waste clearance, processes consistently disrupted in cSVD yet under‐represented in current models. Refinement of *in vitro* platforms to include controlled interstitial flow, perivascular organisation and regulated astrocytic water transport would substantially enhance physiological relevance and enable interrogation of mechanisms that remain inaccessible in traditional models. This approach also provides a tractable framework for identifying and validating targets that modulate fluid clearance pathways. Ultimately, integrating vascular biology, extracellular matrix remodelling, fluid‐dynamic principles and rigorously validated modulators of AQP4 into next‐generation experimental systems will be essential to define the mechanistic basis of cSVD and translate these insights into effective preventive and therapeutic strategies.

## Additional information

## Competing interests

Authors declare they have no competing interests.

## Author contributions

M.M.S. conceptualized the work; S.B. wrote the first draft of the manuscript together with T.S. and L.C.S. S.B. composed the figures; M.M.S., T.S. and L.C.S. edited and supervised the writing and reviewed the manuscript. All authors have read and approved the final version of this manuscript and agree to be accountable for all aspects of the work in ensuring that questions related to the accuracy or integrity of any part of the work are appropriately investigated and resolved. All persons designated as authors qualify for authorship, and all those who qualify for authorship are listed.

## Funding

M.M.S., T.S. and L.C.S. are supported by a Medical Research Council Career Development Award (MR/W027119/1) and by the British Heart Foundation (BHF) and the UK Dementia Research Institute (award number UK DRI‐8203) through UK DRI Ltd, principally funded by the Medical Research Council. M.M.S. acknowledges support from the BHF Centre of Research Excellence, University of Oxford (grant code: RE/24/130024).

## Supporting information


Peer Review History

## Data Availability

No new data were generated or analysed in this article. The data and evidence discussed were derived from previously published studies and publicly available resources cited in the manuscript. Data sharing is therefore not applicable to this article.

## References

[tjp70646-bib-0001] Abbott, J. (2004). Evidence for bulk flow of brain interstitial fluid: Significance for physiology and pathology. Neurochemistry International, 45(4), 545–552. 15186921 10.1016/j.neuint.2003.11.006

[tjp70646-bib-0002] Abbott, N. J. (2005). Dynamics of CNS barriers: Evolution, differentiation, and modulation. Cellular and Molecular Neurobiology, 25(1), 5–23. 15962506 10.1007/s10571-004-1374-y PMC11529509

[tjp70646-bib-0003] Abbott, N. J. , Pizzo, M. E. , Preston, J. E. , Janigro, D. , & Thorne, R. G. (2018). The role of brain barriers in fluid movement in the CNS: Is there a ‘glymphatic’ system? Acta Neuropathologica, 135(3), 387–407. 29428972 10.1007/s00401-018-1812-4

[tjp70646-bib-0004] Al‐Thani, M. , Goodwin‐Trotman, M. , Bell, S. , Patel, K. , Fleming, L. K. , Vilain, C. , Abramowicz, M. , Allan, S. M. , Wang, T. , Cader, M. Z. , Horsburgh, K. , Van Agtmael, T. , Sinha, S. , Markus, H. S. , & Granata, A. (2023). A novel human iPSC model of COL4A1/A2 small vessel disease unveils a key pathogenic role of matrix metalloproteinases. Stem Cell Reports, 18(12), 2386–2399. 37977146 10.1016/j.stemcr.2023.10.014 PMC10724071

[tjp70646-bib-0005] Aldea, R. , Weller, R. O. , Wilcock, D. M. , Carare, R. O. , & Richardson, G. (2019). Cerebrovascular smooth muscle cells as the drivers of intramural periarterial drainage of the brain. Frontiers in Aging Neuroscience, 11, 1. 30740048 10.3389/fnagi.2019.00001 PMC6357927

[tjp70646-bib-0006] Alghanimy, A. , Martin, C. , Gallagher, L. , & Holmes, W. M. (2023). The effect of a novel AQP4 facilitator, TGN‐073, on glymphatic transport captured by diffusion MRI and DCE‐MRI. PLoS ONE, 18(3), e0282955. 36920936 10.1371/journal.pone.0282955 PMC10016657

[tjp70646-bib-0007] Aspelund, A. , Antila, S. , Proulx, S. T. , Karlsen, T. V. , Karaman, S. , Detmar, M. , Wiig, H. , & Alitalo, K. (2015). A dural lymphatic vascular system that drains brain interstitial fluid and macromolecules. Journal of Experimental Medicine, 212(7), 991–999. 26077718 10.1084/jem.20142290 PMC4493418

[tjp70646-bib-0008] Baron‐Menguy, C. , Domenga‐Denier, V. , Ghezali, L. , Faraci, F. M. , & Joutel, A. (2017). Increased Notch3 activity mediates pathological changes in structure of cerebral arteries. Hypertension, 69(1), 60–70. 27821617 10.1161/HYPERTENSIONAHA.116.08015 PMC5145742

[tjp70646-bib-0009] Bohr, T. , Hjorth, P. G. , Holst, S. C. , Hrabětová, S. , Kiviniemi, V. , Lilius, T. , Lundgaard, I. , Mardal, K. A. , Martens, E. A. , Mori, Y. , Nägerl, U. V. , Nicholson, C. , Tannenbaum, A. , Thomas, J. H. , Tithof, J. , Benveniste, H. , Iliff, J. J. , Kelley, D. H. , & Nedergaard, M. (2022). The glymphatic system: Current understanding and modeling. iScience, 25(9), 104987. 36093063 10.1016/j.isci.2022.104987 PMC9460186

[tjp70646-bib-0010] Boster, K. A. S. , Sun, J. , Shang, J. K. , Kelley, D. H. , & Thomas, J. H. (2024). Hydraulic resistance of three‐dimensional pial perivascular spaces in the brain. Fluids Barriers CNS, 21(1), 7. 38212763 10.1186/s12987-023-00505-5 PMC10785473

[tjp70646-bib-0011] Buccellato, F. R. , D'Anca, M. , Serpente, M. , Arighi, A. , & Galimberti, D. (2022). The role of glymphatic system in Alzheimer's and Parkinson's disease pathogenesis. Biomedicines, 10(9), 2261. 36140362 10.3390/biomedicines10092261 PMC9496080

[tjp70646-bib-0012] Campisi, M. , Shin, Y. , Osaki, T. , Hajal, C. , Chiono, V. , & Kamm, R. D. (2018). 3D self‐organized microvascular model of the human blood‐brain barrier with endothelial cells, pericytes and astrocytes. Biomaterials, 180, 117–129. 30032046 10.1016/j.biomaterials.2018.07.014 PMC6201194

[tjp70646-bib-0013] Cao, T. , Yang, C. , Zhang, J. , Yan, Y. , Chen, Z. , Peng, X. , Xia, C. , Pan, M. , Zou, C. , & Lü, T. (2025). The underlying role of pericyte‐related cerebral lymphatic microcirculation dysfunction in cerebral small vessel disease. Neurobiology of Disease, 216, 107101. 40946810 10.1016/j.nbd.2025.107101

[tjp70646-bib-0014] Carare, R. O. , Aldea, R. , Agarwal, N. , Bacskai, B. J. , Bechman, I. , Boche, D. , Bu, G. , Bulters, D. , Clemens, A. , Counts, S. E. , de Leon, M. , Eide, P. K. , Fossati, S. , Greenberg, S. M. , Hamel, E. , Hawkes, C. A. , Koronyo‐Hamaoui, M. , Hainsworth, A. H. , Holtzman, D. , … Verma, A. (2020). Clearance of interstitial fluid (ISF) and CSF (CLIC) group‐part of Vascular Professional Interest area (PIA): Cerebrovascular disease and the failure of elimination of amyloid‐β from the brain and retina with age and Alzheimer's disease‐opportunities for therapy. Alzheimer's & Dementia: Diagnosis, Assessment & Disease Monitoring, 12(1), e12053. 10.1002/dad2.12053 PMC7396859 32775596

[tjp70646-bib-0015] Chagnot, A. , Jaime Garcia, D. , McQuaid, C. , Cholewa‐Waclaw, J. , McDade, K. , Dando, O. , Wardlaw, J. M. , Smith, C. , & Montagne, A. (2026). Abrupt pericyte loss precedes endothelial activation in cerebral small vessel disease. *bioRxiv*. 10.64898/2026.03.19.713028

[tjp70646-bib-0016] Chojdak‐Łukasiewicz, J. , Dziadkowiak, E. , Zimny, A. , & Paradowski, B. (2021). Cerebral small vessel disease: A review. Advances in Clinical and Experimental Medicine: Official Organ Wroclaw Medical University, 30(3), 349–356. 33768739 10.17219/acem/131216

[tjp70646-bib-0017] Collidge, T. A. , Lammie, G. A. , Fleming, S. , & Mullins, J. J. (2004). The role of the renin‐angiotensin system in malignant vascular injury affecting the systemic and cerebral circulations. Progress in Biophysics and Molecular Biology, 84(2–3), 301–319. 14769441 10.1016/j.pbiomolbio.2003.11.003

[tjp70646-bib-0018] Cruz, E. M. , Soares, L. C. , Greene, G. , Messore, F. , Abuelem, M. , Li, M. , Andersen, C. , Domocos, M. , Vitiello, E. , Razak, R. A. , Lawston, M. , Moser, G. , Vasaturo‐Kolodner, T. , Barkat, M. , Bill, R. M. , Mann, E. , Zhou, L. , Salman, M. M. , Bayley, H. , … Szele, F. G. (2026). Astrocyte enrichment of 3D cortical constructs enhances brain repair. Advanced Science, 13(20), e07423. 41744251 10.1002/advs.202507423 PMC13067773

[tjp70646-bib-0019] Cuadrado‐Godia, E. , Dwivedi, P. , Sharma, S. , Ois Santiago, A. , Roquer Gonzalez, J. , Balcells, M. , Laird, J. , Turk, M. , Suri, H. S. , Nicolaides, A. , Saba, L. , Khanna, N. N. , & Suri, J. S. (2018). Cerebral small vessel disease: A review focusing on pathophysiology, biomarkers, and machine learning strategies. Journal of Stroke, 20(3), 302–320. 30309226 10.5853/jos.2017.02922 PMC6186915

[tjp70646-bib-0020] Domocos, M. , Bragin, D. E. , Shanbhag, N. C. , Schlotterose, L. , & Salman, M. M. (2026). Photobiomodulation restores blood‐brain barrier integrity after hypoxia via endothelial von Willebrand factor modulation in a humanised tricellular transwell model. The Journal of Physiology, 604(14), 6080–6103. 10.1113/JP291064 PMC13370692 41965326

[tjp70646-bib-0021] Ferguson, A. C. , Thrippleton, S. , Henshall, D. , Whittaker, E. , Conway, B. , MacLeod, M. , Malik, R. , Rawlik, K. , Tenesa, A. , Sudlow, C. , & Rannikmae, K. (2022). Frequency and phenotype associations of rare variants in 5 monogenic cerebral small vessel disease genes in 200,000 UK biobank participants. Neurology: Genetics, 8(5), e200015. 36035235 10.1212/NXG.0000000000200015 PMC9403885

[tjp70646-bib-0022] Gold, K. A. , Saha, B. , Rajeeva Pandian, N. K. , Walther, B. K. , Palma, J. A. , Jo, J. , Cooke, J. P. , Jain, A. , & Gaharwar, A. K. (2021). 3D Bioprinted multicellular vascular models. Advanced Healthcare Materials, 10(21), e2101141. 34310082 10.1002/adhm.202101141 PMC9295047

[tjp70646-bib-0023] Guey, S. , Lesnik Oberstein, S. A. J. , Tournier‐Lasserve, E. , & Chabriat, H. (2021). Hereditary cerebral small vessel diseases and stroke: A guide for diagnosis and management. Stroke, 52(9), 3025–3032. 34399586 10.1161/STROKEAHA.121.032620

[tjp70646-bib-0024] Hall, C. N. , Reynell, C. , Gesslein, B. , Hamilton, N. B. , Mishra, A. , Sutherland, B. A. , O'Farrell, F. M. , Buchan, A. M. , Lauritzen, M. , & Attwell, D. (2014). Capillary pericytes regulate cerebral blood flow in health and disease. Nature, 508(7494), 55–60. 24670647 10.1038/nature13165 PMC3976267

[tjp70646-bib-0025] Hannawi, Y. , Caceres, E. , Ewees, M. G. , Powell, K. A. , Bratasz, A. , Schwab, J. M. , Rink, C. L. , & Zweier, J. L. (2021). Characterizing the neuroimaging and histopathological correlates of cerebral small vessel disease in spontaneously hypertensive stroke‐prone rats. Frontiers in Neurology, 12, 740298. 34917012 10.3389/fneur.2021.740298 PMC8669961

[tjp70646-bib-0026] Hill, S. A. , Bravo‐Ferrer, I. , Čiulkinytė, A. , Pérez Ramos, N. , Rossetti, I. , Colvin, C. , Beltran‐Lobo, P. , Parra‐Pérez, C. , Emelianova, K. , Dando, O. , Geary, B. , Nirujogi, R. S. , Alessi, D. R. , Lee, D.‐Y. , Lee, Y.‐B. , & Díaz Castro, B. (2025). Molecular profiling of brain endothelial cell to astrocyte endfoot communication in mouse and human. Nature Communications, 16(1), 9750. 10.1038/s41467-025-65487-4 PMC12592424 41198665

[tjp70646-bib-0027] Hodge, R. D. , Bakken, T. E. , Miller, J. A. , Smith, K. A. , Barkan, E. R. , Graybuck, L. T. , Close, J. L. , Long, B. , Johansen, N. , Penn, O. , Yao, Z. , Eggermont, J. , Höllt, T. , Levi, B. P. , Shehata, S. I. , Aevermann, B. , Beller, A. , Bertagnolli, D. , Brouner, K. , … Lein, E. S. (2019). Conserved cell types with divergent features in human versus mouse cortex. Nature, 573(7772), 61–68. 31435019 10.1038/s41586-019-1506-7 PMC6919571

[tjp70646-bib-0028] Hsu, P. D. , Lander, E. S. , & Zhang, F. (2014). Development and applications of CRISPR‐Cas9 for genome engineering. Cell, 157(6), 1262–1278. 24906146 10.1016/j.cell.2014.05.010 PMC4343198

[tjp70646-bib-0029] Hu, X. , Liu, L. , Xiong, M. , & Lu, J. (2024). Application of artificial intelligence‐based magnetic resonance imaging in diagnosis of cerebral small vessel disease. CNS Neuroscience & Therapeutics, 30(7), e14841. 39045778 10.1111/cns.14841 PMC11267174

[tjp70646-bib-0030] Huang, Y. , Clementel, V. , Zhang, M. , Martinez, K. , Spillard, G. , Torres‐Sepulveda, C. , Kisler, K. , Coba, M. P. , & Rust, R. (2026). Multi‐omic profiling reveals pericyte and smooth muscle cell contributions to CADASIL pathology in cell‐specific Notch3 mutant mice. Cell Reports, 45(4), 117285. 41996236 10.1016/j.celrep.2026.117285

[tjp70646-bib-0031] Iliff, J. J. , Lee, H. , Yu, M. , Feng, T. , Logan, J. , Nedergaard, M. , & Benveniste, H. (2013). Brain‐wide pathway for waste clearance captured by contrast‐enhanced MRI. Journal of Clinical Investigation, 123(3), 1299–1309. 23434588 10.1172/JCI67677 PMC3582150

[tjp70646-bib-0032] Iliff, J. J. , Wang, M. , Liao, Y. , Plogg, B. A. , Peng, W. , Gundersen, G. A. , Benveniste, H. , Vates, G. E. , Deane, R. , Goldman, S. A. , Nagelhus, E. A. , & Nedergaard, M. (2012). A paravascular pathway facilitates CSF flow through the brain parenchyma and the clearance of interstitial solutes, including amyloid β. Science Translational Medicine, 4(147), 147ra111. 10.1126/scitranslmed.3003748 PMC3551275 22896675

[tjp70646-bib-0033] Ingber, D. E. (2026). Challenges and opportunities for human organ chips in FDA assessments and pharma pipelines. Cell Stem Cell, 33(2), 176–183. 41564882 10.1016/j.stem.2025.12.022

[tjp70646-bib-0034] Jia, R. , Solé‐Guardia, G. , Verweij, V. , Snabel, J. M. , Geenen, B. , Tuladhar, A. M. , Kleemann, R. , Kiliaan, A. J. , & Wiesmann, M. (2025). Identification and characterization of a translational mouse model for blood‐brain barrier leakage in cerebral small vessel disease. International Journal of Molecular Sciences, 26(14), 6706. 40724955 10.3390/ijms26146706 PMC12294681

[tjp70646-bib-0035] Joutel, A. (2025). The pathobiology of cerebrovascular lesions in CADASIL small vessel disease. Basic & Clinical Pharmacology & Toxicology, 136(5), e70028. 40145673 10.1111/bcpt.70028 PMC11948957

[tjp70646-bib-0036] Joutel, A. , & Faraci, F. M. (2014). Cerebral small vessel disease: Insights and opportunities from mouse models of collagen IV‐related small vessel disease and cerebral autosomal dominant arteriopathy with subcortical infarcts and leukoencephalopathy. Stroke, 45(4), 1215–1221. 24503668 10.1161/STROKEAHA.113.002878 PMC3966958

[tjp70646-bib-0037] Kilpinen, H. , Goncalves, A. , Leha, A. , Afzal, V. , Alasoo, K. , Ashford, S. , Bala, S. , Bensaddek, D. , Casale, F. P. , Culley, O. J. , Danecek, P. , Faulconbridge, A. , Harrison, P. W. , Kathuria, A. , McCarthy, D. , McCarthy, S. A. , Meleckyte, R. , Memari, Y. , Moens, N. , … Gaffney, D. J. (2017). Common genetic variation drives molecular heterogeneity in human iPSCs. Nature, 546(7658), 370–375. 28489815 10.1038/nature22403 PMC5524171

[tjp70646-bib-0038] Kitchen, P. , Salman, M. M. , Halsey, A. M. , Clarke‐Bland, C. , MacDonald, J. A. , Ishida, H. , Vogel, H. J. , Almutiri, S. , Logan, A. , Kreida, S. , Al‐Jubair, T. , Winkel Missel, J. , Gourdon, P. , Törnroth‐Horsefield, S. , Conner, M. T. , Ahmed, Z. , Conner, A. C. , & Bill, R. M. (2020). Targeting aquaporin‐4 subcellular localization to treat central nervous system edema. Cell, 181(4), 784–799.e719. 32413299 10.1016/j.cell.2020.03.037 PMC7242911

[tjp70646-bib-0039] Kremer, R. , Williams, A. , & Wardlaw, J. (2025). Endothelial cells as key players in cerebral small vessel disease. Nature Reviews Neuroscience, 26(3), 179–188. 39743557 10.1038/s41583-024-00892-0

[tjp70646-bib-0040] Lee, D. H. , Lee, E. C. , Park, S. W. , Lee, J. Y. , Lee, M. R. , & Oh, J. S. (2024). Pathogenesis of cerebral small vessel disease: Role of the glymphatic system dysfunction. International Journal of Molecular Sciences, 25(16), 8752. 39201439 10.3390/ijms25168752 PMC11354389

[tjp70646-bib-0041] Lee, S. J. , Zhang, X. , Wu, E. , Sukpraphrute, R. , Sukpraphrute, C. , Ye, A. , & Wang, M. M. (2023). Structural changes in NOTCH3 induced by CADASIL mutations: Role of cysteine and non‐cysteine alterations. Journal of Biological Chemistry, 299(6), 104838. 37209821 10.1016/j.jbc.2023.104838 PMC10318516

[tjp70646-bib-0042] Marazzi, D. , Trovalusci, F. , Di Nardo, P. , & Carotenuto, F. (2025). Organ‐on‐a‐chip and lab‐on‐a‐chip technologies in cardiac tissue engineering. Biomimetics, 11(1), 18. 41589934 10.3390/biomimetics11010018 PMC12838946

[tjp70646-bib-0043] Markou, A. , Unger, L. , Abir‐Awan, M. , Saadallah, A. , Halsey, A. , Balklava, Z. , Conner, M. , Törnroth‐Horsefield, S. , Greenhill, S. D. , Conner, A. , Bill, R. M. , Salman, M. M. , & Kitchen, P. (2022). Molecular mechanisms governing aquaporin relocalisation. Biochimica et Biophysica Acta – Biomembranes, 1864(4), 183853. 34973181 10.1016/j.bbamem.2021.183853 PMC8825993

[tjp70646-bib-0044] McCloskey, M. C. , Ahmad, S. D. , Widom, L. P. , Kasap, P. , Gastfriend, B. D. , Shusta, E. V. , Palecek, S. P. , Engelhardt, B. , Gaborski, T. R. , Flax, J. , Waugh, R. E. , & McGrath, J. L. (2024). Pericytes enrich the basement membrane and reduce neutrophil transmigration in an In vitro model of peripheral inflammation at the blood‐brain barrier. Biomaterials Research, 28, 0081. 39363889 10.34133/bmr.0081 PMC11447289

[tjp70646-bib-0045] Mestre, H. , Tithof, J. , Du, T. , Song, W. , Peng, W. , Sweeney, A. M. , Olveda, G. , Thomas, J. H. , Nedergaard, M. , & Kelley, D. H. (2018). Flow of cerebrospinal fluid is driven by arterial pulsations and is reduced in hypertension. Nature Communications, 9(1), 4878. 10.1038/s41467-018-07318-3 PMC6242982 30451853

[tjp70646-bib-0046] Montagne, A. , Barnes, S. R. , Sweeney, M. D. , Halliday, M. R. , Sagare, A. P. , Zhao, Z. , Toga, A. W. , Jacobs, R. E. , Liu, C. Y. , Amezcua, L. , Harrington, M. G. , Chui, H. C. , Law, M. , & Zlokovic, B. V. (2015). Blood‐brain barrier breakdown in the aging human hippocampus. Neuron, 85(2), 296–302. 25611508 10.1016/j.neuron.2014.12.032 PMC4350773

[tjp70646-bib-0047] Mortensen, K. N. , Sanggaard, S. , Mestre, H. , Lee, H. , Kostrikov, S. , Xavier, A. L. R. , Gjedde, A. , Benveniste, H. , & Nedergaard, M. (2019). Impaired glymphatic transport in spontaneously hypertensive rats. Journal of Neuroscience, 39(32), 6365–6377. 31209176 10.1523/JNEUROSCI.1974-18.2019 PMC6687896

[tjp70646-bib-0048] Mustapha, M. , Nassir, C. , Aminuddin, N. , Safri, A. A. , & Ghazali, M. M. (2019). Cerebral small vessel disease (CSVD) ‐ lessons from the animal models. Frontiers in Physiology, 10, 1317. 31708793 10.3389/fphys.2019.01317 PMC6822570

[tjp70646-bib-0049] Nagelhus, E. A. , & Ottersen, O. P. (2013). Physiological roles of aquaporin‐4 in brain. Physiological Reviews, 93(4), 1543–1562. 24137016 10.1152/physrev.00011.2013 PMC3858210

[tjp70646-bib-0050] Pantoni, L. (2010). Cerebral small vessel disease: From pathogenesis and clinical characteristics to therapeutic challenges. Lancet Neurology, 9(7), 689–701. 20610345 10.1016/S1474-4422(10)70104-6

[tjp70646-bib-0051] Peng, S. , Liu, J. , Liang, C. , Yang, L. , & Wang, G. (2023). Aquaporin‐4 in glymphatic system, and its implication for central nervous system disorders. Neurobiology of Disease, 179, 106035. 36796590 10.1016/j.nbd.2023.106035

[tjp70646-bib-0052] Pierre, A. , Favory, R. , Lancel, S. , & Preau, S. (2025). Can patient‐derived in vitro models improve clinical translation in critical care research when used before animal studies? Intensive Care Medicine Experimental, 13(1), 102. 41091290 10.1186/s40635-025-00814-z PMC12528567

[tjp70646-bib-0053] Pinals, R. L. , & Tsai, L. H. (2022). Building in vitro models of the brain to understand the role of APOE in Alzheimer's disease. Life Science Alliance, 5(11), e202201542. 36167428 10.26508/lsa.202201542 PMC9515460

[tjp70646-bib-0054] Preman, P. , Tcw, J. , Calafate, S. , Snellinx, A. , Alfonso‐Triguero, M. , Corthout, N. , Munck, S. , Thal, D. R. , Goate, A. M. , De Strooper, B. , & Arranz, A. M. (2021). Human iPSC‐derived astrocytes transplanted into the mouse brain undergo morphological changes in response to amyloid‐β plaques. Molecular Neurodegeneration, 16(1), 68. 34563212 10.1186/s13024-021-00487-8 PMC8467145

[tjp70646-bib-0055] Quick, S. , Procter, T. V. , Moss, J. , Seeker, L. , Walton, M. , Lawson, A. , Baker, S. , Beletski, A. , Garcia, D. J. , Mohammad, M. , Mungall, W. , Onishi, A. , Tobola, Z. , Stringer, M. , Jansen, M. A. , Vallatos, A. , Giarratano, Y. , Bernabeu, M. O. , Wardlaw, J. M. , & Williams, A. (2022). Loss of the heterogeneous expression of flippase ATP11B leads to cerebral small vessel disease in a normotensive rat model. Acta Neuropathologica, 144(2), 283–303. 35635573 10.1007/s00401-022-02441-4 PMC9288385

[tjp70646-bib-0056] Rajani, R. M. , Quick, S. , Ruigrok, S. R. , Graham, D. , Harris, S. E. , Verhaaren, B. F. J. , Fornage, M. , Seshadri, S. , Atanur, S. S. , Dominiczak, A. F. , Smith, C. , Wardlaw, J. M. , & Williams, A. (2018). Reversal of endothelial dysfunction reduces white matter vulnerability in cerebral small vessel disease in rats. Science Translational Medicine, 10(448), eaam9507. 29973407 10.1126/scitranslmed.aam9507

[tjp70646-bib-0057] Rasmussen, M. K. , Mestre, H. , & Nedergaard, M. (2022). Fluid transport in the brain. Physiological Reviews, 102(2), 1025–1151. 33949874 10.1152/physrev.00031.2020 PMC8897154

[tjp70646-bib-0058] Richards, A. , van den Maagdenberg, A. M. , Jen, J. C. , Kavanagh, D. , Bertram, P. , Spitzer, D. , Liszewski, M. K. , Barilla‐Labarca, M. L. , Terwindt, G. M. , Kasai, Y. , McLellan, M. , Grand, M. G. , Vanmolkot, K. R. , de Vries, B. , Wan, J. , Kane, M. J. , Mamsa, H. , Schäfer, R. , Stam, A. H. , … Atkinson, J. P. (2007). C‐terminal truncations in human 3'‐5' DNA exonuclease TREX1 cause autosomal dominant retinal vasculopathy with cerebral leukodystrophy. Nature Genetics, 39(9), 1068–1070. 17660820 10.1038/ng2082

[tjp70646-bib-0059] Salman, M. M. , Marsh, G. , Kusters, I. , Delincé, M. , Di Caprio, G. , Upadhyayula, S. , de Nola, G. , Hunt, R. , Ohashi, K. G. , Gray, T. , Shimizu, F. , Sano, Y. , Kanda, T. , Obermeier, B. , & Kirchhausen, T. (2020). Design and validation of a hbuman brain endothelial microvessel‐on‐a‐chip open microfluidic model enabling advanced optical imaging. Frontiers in Bioengineering and Biotechnology, 8, 573775. 33117784 10.3389/fbioe.2020.573775 PMC7576009

[tjp70646-bib-0060] Schröder, H. , Moser, N. , & Huggenberger, S. (2020). The mouse circle of willis. In Neuroanatomy of the Mouse: An Introduction (pp. 333–340). Springer International Publishing.

[tjp70646-bib-0061] Shibata, M. , Ohtani, R. , Ihara, M. , & Tomimoto, H. (2004). White matter lesions and glial activation in a novel mouse model of chronic cerebral hypoperfusion. Stroke, 35(11), 2598–2603. 15472111 10.1161/01.STR.0000143725.19053.60

[tjp70646-bib-0062] Shityakov, S. , & Förster, C. Y. (2018). Computational simulation and modeling of the blood‐brain barrier pathology. Histochemistry and Cell Biology, 149(5), 451–459. 29721642 10.1007/s00418-018-1665-x

[tjp70646-bib-0063] Silasi, G. , She, J. , Boyd, J. D. , Xue, S. , & Murphy, T. H. (2015). A mouse model of small‐vessel disease that produces brain‐wide‐identified microocclusions and regionally selective neuronal injury. Journal of Cerebral Blood Flow and Metabolism, 35(5), 734–738. 25690472 10.1038/jcbfm.2015.8 PMC4420872

[tjp70646-bib-0064] Simard, M. , Arcuino, G. , Takano, T. , Liu, Q. S. , & Nedergaard, M. (2003). Signaling at the gliovascular interface. Journal of Neuroscience, 23(27), 9254–9262. 14534260 10.1523/JNEUROSCI.23-27-09254.2003 PMC6740832

[tjp70646-bib-0065] Singh, A. , Bonnell, G. , De Prey, J. , Buchwald, N. , Eskander, K. , Kincaid, K. J. , & Wilson, C. A. (2023). Small‐vessel disease in the brain. American Heart Journal Plus: Cardiology Research and Practice, 27, 100277. 38511094 10.1016/j.ahjo.2023.100277 PMC10945899

[tjp70646-bib-0066] Singh, V. , Rochakim, N. , Ferraresso, F. , Garg, K. , Choudhury, A. , Kastrup, C. J. , & Ahn, H. J. (2025). Aquaporin‐4 and Caveolin‐1 as mediators of fibrinogen‐driven cerebrovascular pathology in cerebral amyloid angiopathy. bioRxiv. 10.1101/2024.11.11.623066 PMC12812864 41549419

[tjp70646-bib-0067] Smith, A. J. , Yao, X. , Dix, J. A. , Jin, B. J. , & Verkman, A. S. (2017). Test of the ‘glymphatic’ hypothesis demonstrates diffusive and aquaporin‐4‐independent solute transport in rodent brain parenchyma. eLife, 6, e27679. 28826498 10.7554/eLife.27679 PMC5578736

[tjp70646-bib-0068] Smith, E. E. , & Markus, H. S. (2020). New treatment approaches to modify the course of cerebral small vessel diseases. Stroke, 51(1), 38–46. 31752610 10.1161/STROKEAHA.119.024150

[tjp70646-bib-0069] Song, H. W. , Foreman, K. L. , Gastfriend, B. D. , Kuo, J. S. , Palecek, S. P. , & Shusta, E. V. (2020). Transcriptomic comparison of human and mouse brain microvessels. Scientific Reports, 10(1), 12358. 32704093 10.1038/s41598-020-69096-7 PMC7378255

[tjp70646-bib-0070] Soria, G. , Tudela, R. , Márquez‐Martín, A. , Camón, L. , Batalle, D. , Muñoz‐Moreno, E. , Eixarch, E. , Puig, J. , Pedraza, S. , Vila, E. , Prats‐Galino, A. , & Planas, A. M. (2013). The ins and outs of the BCCAo model for chronic hypoperfusion: A multimodal and longitudinal MRI approach. PLoS ONE, 8(9), e74631. 24058609 10.1371/journal.pone.0074631 PMC3776744

[tjp70646-bib-0071] Stringer, M. S. , Lee, H. , Huuskonen, M. T. , MacIntosh, B. J. , Brown, R. , Montagne, A. , Atwi, S. , Ramirez, J. , Jansen, M. A. , Marshall, I. , Black, S. E. , Zlokovic, B. V. , Benveniste, H. , & Wardlaw, J. M. (2021). A review of translational agnetic resonance imaging in human and rodent experimental models of small vessel disease. Translational Stroke Research, 12(1), 15–30. 32936435 10.1007/s12975-020-00843-8 PMC7803876

[tjp70646-bib-0072] Sun, C. , Lin, L. , Yin, L. , Hao, X. , Tian, J. , Zhang, X. , Ren, Y. , Li, C. , & Yang, Y. (2022). Acutely inhibiting AQP4 with TGN‐020 improves functional outcome by attenuating edema and peri‐infarct astrogliosis after cerebral ischemia. Frontiers in Immunology, 13, 870029. 35592320 10.3389/fimmu.2022.870029 PMC9110854

[tjp70646-bib-0073] Šutalo, I. , Bui, A. , Ahmed, S. , Liffman, K. , & Manasseh, R. (2015). Modeling of flow through the circle of willis and cerebral vasculature to assess the effects of changes In the peripheral small cerebral vasculature on the inflows. Engineering Applications of Computational Fluid Mechanics, 8(4), 609–622.

[tjp70646-bib-0074] Syková, E. , & Nicholson, C. (2008). Diffusion in brain extracellular space. Physiological Reviews, 88(4), 1277–1340. 18923183 10.1152/physrev.00027.2007 PMC2785730

[tjp70646-bib-0075] Tang, J. , Zhang, M. , Liu, N. , Xue, Y. , Ren, X. , Huang, Q. , Shi, L. , & Fu, J. (2022). The association between glymphatic system dysfunction and cognitive impairment in cerebral small vessel disease. Frontiers in Aging Neuroscience, 14, 916633. 35813943 10.3389/fnagi.2022.916633 PMC9263395

[tjp70646-bib-0076] Taoka, T. , Masutani, Y. , Kawai, H. , Nakane, T. , Matsuoka, K. , Yasuno, F. , Kishimoto, T. , & Naganawa, S. (2017). Evaluation of glymphatic system activity with the diffusion MR technique: Diffusion tensor image analysis along the perivascular space (DTI‐ALPS) in Alzheimer's disease cases. Japanese Journal of Radiology, 35(4), 172–178. 28197821 10.1007/s11604-017-0617-z

[tjp70646-bib-0077] Taoka, T. , & Naganawa, S. (2021). Imaging for central nervous system (CNS) interstitial fluidopathy: Disorders with impaired interstitial fluid dynamics. Japanese Journal of Radiology, 39(1), 1–14. 32653987 10.1007/s11604-020-01017-0 PMC7813706

[tjp70646-bib-0078] Thomas, J. H. (2019). Fluid dynamics of cerebrospinal fluid flow in perivascular spaces. Journal of the Royal Society, Interface, 16(159), 20190572. 31640500 10.1098/rsif.2019.0572 PMC6833335

[tjp70646-bib-0078a] Tian, M. , Kawaguchi, R. , Shen, Y. , Machnicki, M. , Villegas, N. G. , Cooper, D. R. , Montgomery, N. , Cai, Y. , Haring, J. , Lan, R. , Yuan, A. H. , Williams, C. K. , Magaki, S. , Vinters, H. V. , Zhang, Y. , De Biase, L. M. , Silva, A. J. , & Carmichael, S. T. (2025). Deconstructing the intercellular interactome in vascular dementia with focal ischemia for therapeutic applications. Cell, 188(19), 5157–5174.e20. 40592323 10.1016/j.cell.2025.06.002 PMC12221338

[tjp70646-bib-0079] Unger, L. , Wagner, K. , Steffen, J. H. , Wind, M. L. , Al‐Jubair, T. , Zou, H. , Clarke‐Bland, C. , Murray, R. , Qureshi, B. , Lundström, S. , Gaetani, M. , Poyner, D. , Ayub, H. , Wheatley, M. , Gourdon, P. , Yool, A. J. , Törnroth‐Horsefield, S. , Bill, R. M. , Salman, M. M. , & Kitchen, P. (2024). AER‐270 and TGN‐020 are not aquaporin‐4 water channel blockers. bioRxiv. 10.1101/2024.12.04.625365

[tjp70646-bib-0080] Vanlandewijck, M. , He, L. , Mäe, M. A. , Andrae, J. , Ando, K. , Del Gaudio, F. , Nahar, K. , Lebouvier, T. , Laviña, B. , Gouveia, L. , Sun, Y. , Raschperger, E. , Räsänen, M. , Zarb, Y. , Mochizuki, N. , Keller, A. , Lendahl, U. , & Betsholtz, C. (2018). A molecular atlas of cell types and zonation in the brain vasculature. Nature, 554(7693), 475–480. 29443965 10.1038/nature25739

[tjp70646-bib-0081] Wälchli, T. , Ghobrial, M. , Schwab, M. , Takada, S. , Zhong, H. , Suntharalingham, S. , Vetiska, S. , Gonzalez, D. R. , Wu, R. , Rehrauer, H. , Dinesh, A. , Yu, K. , Chen, E. L. Y. , Bisschop, J. , Farnhammer, F. , Mansur, A. , Kalucka, J. , Tirosh, I. , Regli, L. , … Radovanovic, I. (2024). Single‐cell atlas of the human brain vasculature across development, adulthood and disease. Nature, 632(8025), 603–613. 38987604 10.1038/s41586-024-07493-y PMC11324530

[tjp70646-bib-0082] Wardlaw, J. M. , Debette, S. , Jokinen, H. , De Leeuw, F. E. , Pantoni, L. , Chabriat, H. , Staals, J. , Doubal, F. , Rudilosso, S. , Eppinger, S. , Schilling, S. , Ornello, R. , Enzinger, C. , Cordonnier, C. , Taylor‐Rowan, M. , & Lindgren, A. G. (2021). ESO guideline on covert cerebral small vessel disease. *European Stroke Journal*, 6(2), Cxi–clxii. 10.1177/23969873211012132 PMC8370079 34414301

[tjp70646-bib-0083] Wardlaw, J. M. , Smith, C. , & Dichgans, M. (2013). Mechanisms of sporadic cerebral small vessel disease: Insights from neuroimaging. *The* Lancet Neurology, 12(5), 483–497. 23602162 10.1016/S1474-4422(13)70060-7 PMC3836247

[tjp70646-bib-0084] Wardlaw, J. M. , Smith, C. , & Dichgans, M. (2019). Small vessel disease: Mechanisms and clinical implications. *The* Lancet Neurology, 18(7), 684–696. 31097385 10.1016/S1474-4422(19)30079-1

[tjp70646-bib-0085] Wazny, V. K. , Mahadevan, A. , Nguyen, N. , Wee, H. , Vipin, A. , Lam, T. , Tay, K. Y. , See, J. X. , Sandhu, G. , Leow, Y. J. , D'Agostino, G. , Graf, M. , Sivakumar, A. , Lin, S. , Phuc, N. C. T. , Chen, J. X. Y. , Langley, S. R. , Ang, L. T. , Loh, K. M. , … Cheung, C. (2025). Chronic cerebral hypoperfusion induces venous dysfunction via EPAS1 regulation in mice. Nature Communications, 16(1), 6302. 10.1038/s41467-025-61614-3 PMC12238411 40628749

[tjp70646-bib-0086] Wilms, A. E. , de Boer, I. , & Terwindt, G. M. (2022). Retinal vasculopathy with cerebral leukoencephalopathy and systemic manifestations (RVCL‐S): An update on basic science and clinical perspectives. Cerebral Circulation ‐ Cognition and Behavior, 3, 100046. 36324396 10.1016/j.cccb.2022.100046 PMC9616387

[tjp70646-bib-0087] Yamashiro, M. , Yasutomi, D. , Ohya, Y. , Ohyama, S. , Takashima, H. , & Tokashiki, T. (2025). A case of coexisting heterozygous NOTCH3 and HTRA1 mutations in cerebral small vessel disease. Human Genome Variation, 12(1), 13. 40634305 10.1038/s41439-025-00317-z PMC12241498

[tjp70646-bib-0088] Yamori, Y. , Horie, R. , Handa, H. , Sato, M. , & Fukase, M. (1976). Pathogenetic similarity of strokes in stroke‐prone spontaneously hypertensive rats and humans. Stroke, 7(1), 46–53. 1258104 10.1161/01.str.7.1.46

[tjp70646-bib-0089] Yamori, Y. , Sagara, M. , Mori, H. , Mori, M. , & Group, C. S. (2022). Stroke‐prone SHR as experimental models for cardiovascular disease risk reduction in humans. Biomedicines, 10(11), 2974. 36428542 10.3390/biomedicines10112974 PMC9687971

[tjp70646-bib-0090] Yang, A. C. , Vest, R. T. , Kern, F. , Lee, D. P. , Agam, M. , Maat, C. A. , Losada, P. M. , Chen, M. B. , Schaum, N. , Khoury, N. , Toland, A. , Calcuttawala, K. , Shin, H. , Pálovics, R. , Shin, A. , Wang, E. Y. , Luo, J. , Gate, D. , Schulz‐Schaeffer, W. J. , … Wyss‐Coray, T. (2022). A human brain vascular atlas reveals diverse mediators of Alzheimer's risk. Nature, 603(7903), 885–892. 35165441 10.1038/s41586-021-04369-3 PMC9635042

[tjp70646-bib-0091] Yuan, L. , Chen, X. , Jankovic, J. , & Deng, H. (2024). CADASIL: A NOTCH3‐associated cerebral small vessel disease. Journal of Advanced Research, 66, 223–235. 38176524 10.1016/j.jare.2024.01.001 PMC11674792

[tjp70646-bib-0092] Zhang, C. , Zheng, H. , Li, X. , Li, S. , Li, W. , Wang, Z. , Niu, S. , Wang, X. , & Zhang, Z. (2022). Novel mutations in HTRA1‐related cerebral small vessel disease and comparison with CADASIL. Annals of Clinical and Translational Neurology, 9(10), 1586–1595. 36047879 10.1002/acn3.51654 PMC9539375

