# Supplementary material for: Experimental models of cerebral small vessel disease: Physiological constraints, translational challenges and future directions
Source: J Physiol. 2026 Jun 5;604(14):5671–89. doi: 10.1113/JP290165 (PMC13370708; doi:10.1113/JP290165)
Supplement: Supplementary file 1 — Peer Review History [file TJP-604-5671-s001.pdf]

The Journal of Physiology

Experimental models of cerebral small vessel disease: physiological constraints, translational challenges and future directions

Sophie Beaumont, Tanya Singh, Luana Campos Soares, and Mootaz M. Salman
DOI: 10.1113/JP290165

Corresponding author(s): Mootaz Salman (mootaz.salman@dpag.ox.ac.uk)

The referees have opted to remain anonymous.

Review Timeline:

Submission Date:	22-Feb-2026
Editorial Decision:	01-Apr-2026
Revision Received:	28-Apr-2026
Accepted:	19-May-2026

Senior Editor: Laura Bennet

Reviewing Editor: Valentina Mosienko

Transaction Report:

(Note: With the exception of the correction of typographical or spelling errors that could be a source of ambiguity, letters and reports are not edited. Depending on transfer agreements, referee reports obtained elsewhere may or may not be included in this compilation. Referee reports are anonymous unless the Referee chooses to sign their reports.)

Dear Dr Salman,

Re: JP-PL-2026-290165 "**Bayliss-Starling Prize Lecture 2025: Experimental models of cerebral small vessel disease: physiological constraints, translational challenges and future directions**" by Sophie Beaumont, Tanya Singh, Luana C. Soares, and Mootaz M. Salman

Thank you for submitting your manuscript to The Journal of Physiology. It has been assessed by a Reviewing Editor and by 2 expert referees and we are pleased to tell you that it is acceptable for publication following satisfactory revision.

Please advise your co-authors of this decision as soon as possible.

The referee reports are copied at the end of this email.

Please address all the points raised and incorporate all requested revisions or explain in your Response to Referees why a change has not been made. We hope you will find the comments helpful and that you will be able to return your revised manuscript within 4 weeks. If you require longer than this, please contact journal staff: jp@physoc.org.

Your revised manuscript should be submitted online using the link in your Author Tasks Link Not Available. This link is accessible via your account as Corresponding Author; it is not available to your co-authors. If this presents a problem, please contact journal staff (jp@physoc.org). Image files from the previous version are retained on the system. Please ensure you replace or remove any files that are being revised.

If you do not wish to submit a revised version of your manuscript, you must inform our journal staff (jp@physoc.org) or reply to this email to request withdrawal. Please note that a manuscript must be formally withdrawn from the peer review process at one journal before it may be submitted to another journal.

TRANSPARENT PEER REVIEW POLICY: To improve the transparency of its peer review process The Journal of Physiology publishes online, as supporting information, the peer review history of all articles accepted for publication. Readers will have access to decision letters, including Editors' comments and referee reports, for each version of the manuscript, as well as any author responses to peer review comments. Referees can decide whether or not they wish to be named on the peer review history document.

LANGUAGE EDITING AND SUPPORT FOR PUBLICATION: If you would like help with English language editing, or other article preparation support, Wiley Editing Services offers expert help, including English Language Editing, as well as translation, manuscript formatting, and figure formatting at www.wileyauthors.com/eeo/preparation. You can also find resources for Preparing Your Article for general guidance about writing and preparing your manuscript at www.wileyauthors.com/eeo/prepresources.

REVISION CHECKLIST:
Upload a full Response to Referees file. To create your 'Response to Referees' copy all the reports, including any comments from the Senior and Reviewing Editors, into a Microsoft Word, or similar, file and respond to each point, using font or background colour to distinguish comments and responses and upload as the required file type.

Please upload two versions of your manuscript text: one with all relevant changes highlighted and one clean version with no changes tracked. The manuscript file should include all tables and figure legends, but each figure/graph should be uploaded as separate, high-resolution files.

You may also upload:
- 'Potential Cover Art' for consideration as the issue's cover image
- Appropriate Supporting Information (Video, audio or data set: see https://jp.msubmit.net/cgi-bin/main.plex?form_type=display_requirements#supp).

We look forward to receiving your revised submission.

If you have any queries, please reply to this email and we will be pleased to advise.

Yours sincerely,

Laura Bennet
Senior Editor
The Journal of Physiology

REQUIRED ITEMS:

- Your MS must include a complete "Additional information section" with the following 4 headings and content:

Competing Interests: A statement regarding competing interests. If there are no competing interests, a statement to this effect must be included. All authors should disclose any conflict of interest in accordance with journal policy.

Author contributions: Each author should take responsibility for a particular section of the study and have contributed to writing the paper. Acquisition of funding, administrative support or the collection of data alone does not justify authorship; these contributions to the study should be listed in the Acknowledgements. Additional information such as 'X and Y have contributed equally to this work' may be added as a footnote on the title page.

It must be stated that all authors approved the final version of the manuscript and that all persons designated as authors qualify for authorship, and all those who qualify for authorship are listed.

Funding: Authors must indicate all sources of funding, including grant numbers. If authors have not received funding, this must be stated.

It is the responsibility of authors funded by RCUK to adhere to their policy regarding funding sources and underlying research material. The policy requires funding information to be included within the acknowledgement section of a paper. Guidance on how to acknowledge funding information is provided by the Research Information Network. The policy also requires all research papers, if applicable, to include a statement on how any underlying research materials, such as data, samples or models, can be accessed. However, the policy does not require that the data must be made open. If there are considered to be good or compelling reasons to protect access to the data, for example commercial confidentiality or legitimate sensitivities around data derived from potentially identifiable human participants, these should be included in the statement.

Acknowledgements: Acknowledgements should be the minimum consistent with courtesy. The wording of acknowledgements of scientific assistance or advice must have been seen and approved by the persons concerned. This section should not include details of funding.

- Please upload separate high quality figure files via the submission form.

- Author profile(s) must be uploaded via the submission form. Authors should submit a short biography (no more than 100 words for one author or 150 words in total for two authors) and a portrait photograph of the two leading authors on the paper. These should be uploaded and clearly labelled together in a Word document with the revised version of the manuscript. Any standard image format for the photograph is acceptable, but the resolution should be at least 300 DPI and preferably more. A group photograph of all authors is also acceptable, providing the biography for the whole group does not exceed 150 words.

- Please ensure that the Article File you upload is a Word file.

EDITOR COMMENTS

Reviewing Editor:

Thank you for submitting your work to the Journal of Physiology. Both reviewers provided very positive evaluations of your manuscript, particularly highlighting its timeliness and comprehensive nature. They have also raised a number of points that should be addressed before the manuscript can be accepted. Please ensure that you respond carefully to all reviewer comments during your revision.

REFEREE COMMENTS

Referee #1:

This article entitled "Experimental models of cerebral small vessel disease: physiological constraints, translational challenges and future directions" provides an overview of experimental approaches currently used to study the mechanisms underlying cerebral small vessel disease (cSVD). The authors summarise the strengths and limitations of several model

systems and discuss how these approaches can inform our understanding of disease mechanisms and therapeutic development.

The manuscript covers a range of experimental paradigms, including genetic animal models, vascular injury or hypoperfusion models, as well as emerging cellular and in vitro systems. The authors highlight how these models reproduce specific aspects of cSVD pathology while acknowledging limitations that may affect their physiological relevance and translational value.

The topic is timely given the increasing recognition that no single experimental model captures the complexity of human cSVD. Overall, the review provides a useful synthesis of the available experimental systems and the challenges associated with translating findings to human disease. The overview of experimental models and their limitations may be useful for researchers entering the field or seeking a broad perspective on methodological approaches used in cSVD research.

Major Comments

1. The title suggests that the manuscript will address experimental models of cSVD, physiological constraints, and translational challenges. While the review provides a comprehensive overview of experimental models, the discussion of physiological constraints and translational considerations is comparatively less developed.

Expanding the discussion on how experimental models differ from human cerebrovascular physiology, and how these limitations influence translational interpretation, would strengthen the manuscript and better align the content with the title.

2. The manuscript discusses both in vitro and in vivo systems, but the balance between these approaches could be clarified. In particular, the distinct roles of cellular models and animal models in studying cSVD mechanisms could be discussed more explicitly.

While in vitro platforms are valuable for mechanistic studies, many aspects of cSVD (e.g., vascular remodelling, haemodynamic alterations, and long-term neurovascular interactions) are inherently multicellular and systemic processes. A clearer discussion of how in vivo models capture these physiological processes would strengthen the translational perspective of the review.

3. Although vascular pathology and endothelial dysfunction are discussed, the broader neurogliovascular unit could be more systematically integrated. cSVD involves interactions between endothelial cells, vascular smooth muscle cells, pericytes, astrocytes, and neurons, and these interactions are central to disease progression.

A more structured framework describing how different experimental models capture dysfunction within the neurogliovascular unit would improve the conceptual clarity of the manuscript.

4. The manuscript discusses glymphatic and perivascular clearance pathways in detail, but the role of blood-brain barrier dysfunction and capillary-level pathology appears comparatively underdeveloped. Given that cSVD primarily affects small penetrating vessels and capillary networks, where endothelial dysfunction and BBB alterations are increasingly recognised as key features, a more balanced discussion of capillary-level mechanisms would strengthen the manuscript. In particular, pericytes and their interactions with endothelial cells, which are important for microvascular stability and BBB integrity, receive relatively limited attention. Expanding this aspect would provide a more comprehensive view of microvascular dysfunction in cSVD.

5. The manuscript highlights translational challenges but could further develop criteria for evaluating the translational relevance of experimental models. For example, models could be discussed in relation to whether they reproduce key features of human cSVD, such as:

- microvascular structural alterations

- white matter injury

- BBB dysfunction

- altered cerebral blood flow regulation

Providing such a framework would make the review particularly useful for researchers selecting appropriate experimental models.

Minor Comments

- The manuscript occasionally uses terms such as microvascular dysfunction, small vessel disease, and microangiopathy. Clarifying these terms early in the manuscript would improve readability.

- The future directions section could be expanded to highlight emerging approaches such as advanced vascular imaging, multi-omics strategies, and improved human-based modelling systems.

- Including several additional recent references on experimental cSVD models and neurogliovascular dysfunction may strengthen the literature context.

- Some sentences could be streamlined for clarity, particularly in sections where multiple models are described sequentially.

Referee #2:

This is a timely review in an area of high research interest, it is well suited to the Journal of Physiology and I expect it will be of interest to a wide readership. The underlying mechanisms of cSVD remain incompletely understood and, as highlighted in the current review, progress can be limited by the availability of suitable models and technical approaches. As no model can fully recapitulate human SVD, all (whether in vivo, in vitro, or in silico) will inherently have strengths and weaknesses which must be considered when selecting the appropriate model for specific research questions. This manuscript provides a high level overview of various in vivo, in vitro and in silico models commonly used in cSVD research, highlighting the pathological features which they replicate and offering relevant considerations which should be taken into account when selecting to use each model. The review then argues that dysfunction of the glymphatic system should be considered as an underlying pathology in several diseases including cSVD, potentially providing a novel therapeutic target. The key points of the review lie in highlighting the strength of in vitro approaches to enable well-controlled experiments in this field; the need to further develop such approaches so that they better recapitulate human cSVD conditions thus providing better insight into the mechanisms of cSVD; and the need to consider glymphatic system dysfunction as a feature of cSVD comorbidity. The review is well written with points clearly and concisely made.

Minor comments:

- Although there is relatively limited information provided related to in silico models of cSVD, it seems that core points have been covered.

- Paragraph about EC vulnerability (second paragraph of Rodent Models section): it's unclear to a reader how EC dysfunction is linked to CADASIL and mutations in the Notch3 receptor (which is expressed predominantly in vascular smooth muscle). The link should be made clearer or these ideas could be clearly separated into two points (e.g. separate paragraphs).

- Paragraph 1 of Rodent Models section mentions the ATP11BKO mouse model and refers the reader to Table 1. Table 1 describes an ATP11BKO rat model.

- Table 1 - Thy1-GFP tg mouse (Silasi et al 2015): Endovascular injection of fluorescent microspheres produces microocclusions. Applying this technique in Thy1-GFP mice, Silasi et al. examined the impact of the microocclusions on neuronal structure. Given the cSVD model is the focus of this review and of Table 1, I would suggest that the authors consider listing the model as injection of fluorescent beads which can, I assume, be applied to any mouse model. The Thy1-GFP model alone is not a model of cSVD. The authors could mention the use of Thy1-GFP line to allow investigation of neuronal structural changes.

- In addition to the BCCAO model, the authors may wish to also include the BCAS model, commonly used in the field (in mice). In the BCAS model microcoils create gradual narrowing of vessels, leading to a chronic moderate reduction in CBF. Compared to BCCAO, BCAS may be better suited for white matter-focussed studies and chronic reduction of CBF. In addition to reproducing aspects of cSVD, the BCAS model is of particular interest for subcortical ischemic vascular dementia, which may be why the authors have chosen not to include it in the current review? In which case, I leave it to them to determine whether it's inclusion represents a useful addition to the review.

- A final proofread would pick up the remaining few typographical errors, for example, mis-definition of IPAD. Should this be intramural periarterial drainage, rather than intramural periarterial damage?

- Figure 2: To aid the reader it would be useful to define abbreviations in the figure legend. I found it less clear to understand how (4) links to the rest of the diagram, and to understand from the figure that fluid can drain through the arachnoid granulation to the blood. These details were well described in the legend but I found this part of the diagram more difficult to interpret.

END OF COMMENTS

REFEREE COMMENTS

We thank the referees for their careful and insightful evaluation of this work. Their comments have prompted a substantial refinement of the manuscript, shifting it from a descriptive synthesis of experimental models towards a mechanistically grounded and translationally oriented framework for studying cerebral small vessel disease (cSVD). In particular, the revised version more explicitly integrates neurovascular unit biology, capillary-level pathology and brain clearance mechanisms, and introduces a structured approach to evaluating physiological fidelity across model systems. Responses to each comment are provided below in blue, with revised text indicated in italics. We believe these changes significantly strengthen the conceptual contribution of the review and its relevance to both mechanistic research and therapeutic development.

Referee #1: This article entitled "Experimental models of cerebral small vessel disease: physiological constraints, translational challenges and future directions" provides an overview of experimental approaches currently used to study the mechanisms underlying cerebral small vessel disease (cSVD). The authors summarise the strengths and limitations of several model systems and discuss how these approaches can inform our understanding of disease mechanisms and therapeutic development. The manuscript covers a range of experimental paradigms, including genetic animal models, vascular injury or hypoperfusion models, as well as emerging cellular and in vitro systems. The authors highlight how these models reproduce specific aspects of cSVD pathology while acknowledging limitations that may affect their physiological relevance and translational value. The topic is timely given the increasing recognition that no single experimental model captures the complexity of human cSVD. Overall, the review provides a useful synthesis of the available experimental systems and the challenges associated with translating findings to human disease. The overview of experimental models and their limitations may be useful for researchers entering the field or seeking a broad perspective on methodological approaches used in cSVD research.

Major Comments

1. The title suggests that the manuscript will address experimental models of cSVD, physiological constraints, and translational challenges. While the review provides a comprehensive overview of experimental models, the discussion of physiological constraints and translational considerations is comparatively less developed. Expanding the discussion on how experimental models differ from human cerebrovascular physiology, and how these limitations influence translational interpretation, would strengthen the manuscript and better align the content with the title.

We thank the reviewer for this important observation. We agree that the treatment of physiological constraints and translational interpretation was not sufficiently developed relative to the scope implied by the title.

We have therefore expanded the *Conclusions and future directions* section to explicitly define how experimental systems diverge from human cerebrovascular physiology and how these differences constrain mechanistic interpretation and translation. This revision reframes the manuscript from a descriptive synthesis of models to a framework for evaluating their physiological fidelity and translational utility.

2. The manuscript discusses both in vitro and in vivo systems, but the balance between these approaches could be clarified. In particular, the distinct roles of cellular models and animal models in studying cSVD mechanisms could be discussed more explicitly. While in vitro platforms are valuable for mechanistic studies, many aspects of cSVD (e.g., vascular remodelling, haemodynamic alterations, and long-term neurovascular interactions) are inherently multicellular and systemic processes. A clearer discussion of how in vivo models capture these physiological processes would strengthen the translational perspective of the review.

We agree that the complementary roles of in vitro and in vivo systems required clearer articulation. The manuscript has been revised to explicitly distinguish these roles. In vitro platforms are now positioned as enabling causal, cell-type-specific interrogation under controlled conditions, whereas in vivo models are defined as essential for capturing haemodynamic regulation, vascular remodelling, and chronic neurovascular interactions. The revised text emphasises that robust mechanistic inference in cSVD requires convergence across these systems, rather than prioritisation of either approach in isolation.

3. Although vascular pathology and endothelial dysfunction are discussed, the broader neurogliovascular unit could be more systematically integrated. cSVD involves interactions between endothelial cells, vascular smooth muscle cells, pericytes, astrocytes, and neurons, and these interactions are central to disease progression. A more structured framework describing how different experimental models capture dysfunction within the neurogliovascular unit would improve the conceptual clarity of the manuscript.

We thank the reviewer for highlighting this conceptual gap. We agree that explicit integration of the neurovascular unit (NVU) is essential for mechanistic clarity.

We have addressed this through three coordinated revisions. First, we strengthened the representation of cell-type–specific mechanisms across sections, including VSMC-driven

pathology in rodent models and pericyte-dependent regulation in in vitro systems. Second, we introduced a dedicated section, Interactions at the capillary level, which anchors cSVD within the NVU framework. Third, we clarified that cSVD pathology emerges from disruption of coordinated multicellular signalling rather than isolated cellular dysfunction.

Together, these changes provide a coherent framework linking endothelial, mural, glial and neuronal contributions to disease progression.

Rodent models:

"A complementary body of research highlights the role of vascular smooth muscle cell (VSMC) dysfunction in cSVD pathogenesis. […] Consistent with this, recent conditional knock-in models demonstrate that pericyte- and VSMC-specific expression of the *Notch3*^{R170C} variant independently recapitulates key CADASIL features, but through divergent pathways, with cell type–specific vascular, neuroinflammatory and proteomic signatures.(Huang et al., 2026).*"*

Considering the importance of this question, we have also added the following sections: Interactions at the capillary level

"cSVD is fundamentally a disorder of the microvasculature, yet most experimental models remain reductionist, focusing on isolated cell types or discrete pathological features. The small arterioles and capillaries affected in cSVD constitute the anatomical and functional core of the neurovascular unit (NVU), an integrated system comprising endothelial cells, vascular smooth muscle cells (VSMCs), pericytes, astrocytes and neurons that collectively regulate cerebral blood flow, BBB integrity and interstitial fluid dynamics […] Using single-nucleus RNA sequencing across endothelial cells, pericytes, VSMCs, astrocytes, microglia and neurons, they reconstructed the NVU interactome at single-cell resolution. Their findings demonstrate that vascular pathology cannot be attributed to a single cellular compartment, but instead emerges from the disruption of coordinated multicellular signalling across the NVU."

"In vitro, multicellular co-culture systems incorporating iPSC-derived endothelial cells, pericytes and astrocytes have demonstrated that BBB integrity is an emergent property of coordinated signalling across multiple cell types, rather than an intrinsic feature of the endothelium alone (Ferguson et al., 2022). Inclusion of pericytes in microphysiological BBB models, for example, modulates endothelial responses under inflammatory conditions, including leukocyte transmigration, in ways that cannot be predicted from endothelial monocultures (McCloskey et al., 2024) […] Moving forward, experimental platforms should aim to reconstruct the NVU as an integrated system, capturing pericyte–endothelial,

astrocyte-endothelial and broader neurovascular interfaces central to cSVD pathophysiology. Coupling such systems with single-cell transcriptomic resolution will be critical to resolve cell type–specific contributions within this multicellular network."

4. The manuscript discusses glymphatic and perivascular clearance pathways in detail, but the role of blood brain barrier dysfunction and capillary-level pathology appears comparatively underdeveloped. Given that cSVD primarily affects small penetrating vessels and capillary networks, where endothelial dysfunction and BBB alterations are increasingly recognised as key features, a more balanced discussion of capillary-level mechanisms would strengthen the manuscript. In particular, pericytes and their interactions with endothelial cells, which are important for microvascular stability and BBB integrity, receive relatively limited attention. Expanding this aspect would provide a more comprehensive view of microvascular dysfunction in cSVD.

We agree that capillary-level mechanisms and pericyte biology were underrepresented.

This has been addressed by expanding both the In vitro models section and the new Interactions at the capillary level section. The revised text now emphasises pericytes as active regulators of capillary dynamics and endothelial behaviour, rather than purely structural components. In particular, we highlight their role in modulating vascular stability, inflammatory signalling, and leukocyte trafficking, processes directly relevant to cSVD progression.

We also included more detail on pericytes in the "Interactions at the Capillary Level" section:

"In vitro, multicellular co-culture systems incorporating iPSC-derived endothelial cells, pericytes and astrocytes have demonstrated that BBB integrity is an emergent property of coordinated signalling across multiple cell types, rather than an intrinsic feature of the endothelium alone (Ferguson et al., 2022). Inclusion of pericytes in microphysiological BBB models, for example, modulates endothelial responses under inflammatory conditions, including leukocyte transmigration, in ways that cannot be predicted from endothelial monocultures (McCloskey et al., 2024)."

These revisions provide a more balanced and mechanistically grounded view of microvascular dysfunction.

5. The manuscript highlights translational challenges but could further develop criteria for evaluating the translational relevance of experimental models. For example, models could be discussed in relation to whether they reproduce key features of human cSVD, such as:- microvascular structural alterations- white matter injury- BBB dysfunction- altered cerebral

blood flow regulation. Providing such a framework would make the review particularly useful for researchers selecting appropriate experimental models.

We thank the reviewer for this constructive suggestion. We agree that an explicit framework for evaluating translational relevance is critical.

We have therefore introduced a Translational Relevance column in Table 3, structured around four key disease features: microvascular structural alterations, white matter injury, BBB dysfunction, and cerebral blood flow regulation. This provides a systematic basis for comparing experimental systems and shifts the manuscript towards a more decision-oriented framework for model selection.

Minor Comments

- The manuscript occasionally uses terms such as microvascular dysfunction, small vessel disease, and microangiopathy. Clarifying these terms early in the manuscript would improve readability.

We agree that early clarification of terminology improves conceptual precision. The Introduction has been revised to define cSVD explicitly as cerebral microangiopathy and to standardise terminology across the manuscript.

"Also referred to as cerebral microangiopathy, cSVD is characterised by dysfunction of the brain's small penetrating arteries, arterioles, capillaries and venules, with consequent impairment of endothelial and blood-brain barrier (BBB)integrity that ultimately drives injury to both white and grey matter (Kremer et al., 2025)"

- The future directions section could be expanded to highlight emerging approaches such as advanced vascular imaging, multi-omics strategies, and improved human-based modelling systems.

We have expanded the Conclusions and future directions section to incorporate emerging approaches, including human iPSC-derived neurovascular models, multi-omics integration, and high-resolution vascular imaging. These additions strengthen the forward-looking and translational aspects of the manuscript.

"Human-relevant systems, including patient-specific iPSC-derived neurovascular models, combined with multi-omics approaches integrating transcriptomic, proteomic and epigenetic readouts, offer a level of mechanistic resolution not previously achievable. Parallel advances in high-resolution vascular imaging, including 7T MRI and two-photon microscopy, will be essential to validate experimental findings and bridge the gap between model systems and human disease."

- Including several additional recent references on experimental cSVD models and neurogliovascular dysfunction may strengthen the literature context.

We have expanded the literature base to incorporate recent advances in cSVD modelling, neurovascular unit biology, pericyte function, and glymphatic and clearance mechanisms.

- Some sentences could be streamlined for clarity, particularly in sections where multiple models are described sequentially.

The manuscript has undergone a full editorial revision. Redundant material has been removed, sections describing multiple models have been streamlined, and transitions have been clarified to improve readability and logical flow.

Referee #2: This is a timely review in an area of high research interest, it is well suited to the Journal of Physiology and I expect it will be of interest to a wide readership. The underlying mechanisms of cSVD remain incompletely understood and, as highlighted in the current review, progress can be limited by the availability of suitable models and technical approaches. As no model can fully recapitulate human SVD, all (whether in vivo, in vitro, or in silico) will inherently have strengths and weaknesses which must be considered when selecting the appropriate model for specific research questions. This manuscript provides a high level overview of various in vivo, in vitro and in silico models commonly used in cSVD research, highlighting the pathological features which they replicate and offering relevant considerations which should be taken into account when selecting to use each model. The review then argues that dysfunction of the glymphatic system should be considered as an underlying pathology in several diseases including cSVD, potentially providing a novel therapeutic target. The key points of the review lie in highlighting the strength of in vitro approaches to enable well-controlled experiments in this field; the need to further develop such approaches so that they better recapitulate human cSVD conditions thus providing better insight into the mechanisms of cSVD; and the need to consider glymphatic system dysfunction as a feature of cSVD comorbidity. The review is well written with points clearly and concisely made.

Minor comments:

- Although there is relatively limited information provided related to in silico models of cSVD, it seems that core points have been covered.

We thank the reviewer for their positive and constructive assessment.

- Paragraph about EC vulnerability (second paragraph of Rodent Models section): it's unclear to a reader how EC dysfunction is linked to CADASIL and mutations in the Notch3 receptor (which is expressed predominantly in vascular smooth muscle). The link should be made clearer or these ideas could be clearly separated into two points (e.g. separate paragraphs).

We agree that the original text did not sufficiently distinguish these mechanisms.

We have separated endothelial-driven and VSMC-driven pathways into distinct paragraphs in the Rodent models section. This clarifies that ATP11B-associated endothelial dysfunction and NOTCH3-driven VSMC pathology represent mechanistically distinct, though convergent, routes to cSVD-like pathology.

- Paragraph 1 of Rodent Models section mentions the ATP11BKO mouse model and refers the reader to Table 1. Table 1 describes an ATP11BKO rat model.

This has been corrected. The manuscript now consistently refers to the ATP11BKO rat model in line with the original publication.

- Table 1 - Thy1-GFP tg mouse (Silasi et al 2015): Endovascular injection of fluorescent microspheres produces microocclusions. Applying this technique in Thy1-GFP mice, Silasi et al. examined the impact of the microocclusions on neuronal structure. Given the cSVD model is the focus of this review and of Table 1, I would suggest that the authors consider listing the model as injection of fluorescent beads which can, I assume, be applied to any mouse model. The Thy1-GFP model alone is not a model of cSVD. The authors could mention the use of Thy1-GFP line to allow investigation of neuronal structural changes.

We have revised the description to distinguish between the experimental technique (endovascular microsphere injection) and the use of the Thy1-GFP line for neuronal visualisation. This avoids misclassification of the transgenic line as a cSVD model.

- In addition to the BCCAO model, the authors may wish to also include the BCAS model, commonly used in the field (in mice). In the BCAS model microcoils create gradual narrowing of vessels, leading to a chronic moderate reduction in CBF. Compared to BCCAO, BCAS may be better suited for white matter-focussed studies and chronic reduction of CBF. In addition to reproducing aspects of cSVD, the BCAS model is of particular interest for subcortical ischemic vascular dementia, which may be why the authors have chosen not to include it in the current review? In which case, I leave it to them to determine whether it's inclusion represents a useful addition to the review.

We agree that the BCAS model is relevant and have incorporated it into Table 1. Its suitability for modelling chronic hypoperfusion and white matter pathology is now explicitly stated.

- A final proofread would pick up the remaining few typographical errors, for example, mis-definition of IPAD. Should this be intramural periarterial drainage, rather than intramural periarterial damage?

We thank the reviewer for spotting this. IPAD has been corrected to intramural periarterial drainage, and the manuscript has been thoroughly proofread.

- Figure 2: To aid the reader it would be useful to define abbreviations in the figure legend. I found it less clear to understand how (4) links to the rest of the diagram, and to understand from the figure that fluid can drain through the arachnoid granulation to the blood. These details were well described in the legend but I found this part of the diagram more difficult to interpret.

We have revised both the figure and legend to clarify the drainage pathway, including the role of arachnoid granulations in fluid clearance to the bloodstream. The figure and its legend have been revised to clarify the drainage pathway shown in panel (4), including the role of the arachnoid granulation in facilitating fluid drainage to the bloodstream.

Dear Editor,

We are pleased to submit the revised version of our manuscript entitled "Experimental models of cerebral small vessel disease: physiological constraints, translational challenges and future directions."

Cerebral small vessel disease (cSVD) remains a major and growing contributor to stroke and dementia, yet mechanistic understanding has lagged behind its clinical impact. A key barrier has been the absence of experimental systems that capture the integrated, multicellular and slowly progressive nature of the disease. While a wide range of in vivo, in vitro and computational models exist, their physiological fidelity and translational relevance are rarely evaluated within a unified framework.

In this review, we move beyond a descriptive catalogue of model systems to propose a mechanistically grounded perspective on cSVD modelling. We critically assess how current approaches capture, or fail to capture, key aspects of human cerebrovascular physiology, and we identify the neurovascular unit and brain clearance pathways as central, yet underrepresented, axes of disease modelling. By integrating advances in multicellular in vitro systems, organ-on-chip platforms, and emerging glymphatic concepts, we outline a path towards next-generation models capable of resolving causal mechanisms and supporting therapeutic discovery.

Following the referees' comments, the manuscript has been substantially revised. We have strengthened the discussion of physiological constraints and translational interpretation, introduced a structured framework for evaluating model relevance, and expanded integration of neurovascular unit biology and capillary-level mechanisms. These revisions sharpen the conceptual focus of the review and position it as a resource for guiding both experimental design and translational strategy in cSVD research.

We believe the revised manuscript will be of broad interest to researchers working across cerebrovascular biology, neurodegeneration, and experimental modelling, and that it provides a timely and necessary framework for advancing the field.

Best wishes,

Mootaz Salman

On behalf of all the authors

Dear Dr Salman,

Re: JP-PL-2026-290165R1 "**Experimental models of cerebral small vessel disease: physiological constraints, translational challenges and future directions**" by Sophie Beaumont, Tanya Singh, Luana Campos Soares, and Mootaz M. Salman

We are pleased to tell you that your paper has been accepted for publication in The Journal of Physiology.

TRANSPARENT PEER REVIEW POLICY: To improve the transparency of its peer review process The Journal of Physiology publishes online, as supporting information, the peer review history of all articles accepted for publication. Readers will have access to decision letters, including Editors' comments and referee reports, for each version of the manuscript, as well as any author responses to peer review comments. Referees can decide whether or not they wish to be named on the peer review history document.

The last Word (or similar) version of the manuscript provided will be used by the Production Editor to prepare your proof. When this is ready you will receive an email containing a link to Wiley's Online Proofing System. The proof should be thoroughly checked and corrected as promptly as possible.

Authors should note that it is too late at this point to offer corrections prior to proofing. The accepted version will be published online, ahead of the copy edited and typeset version being made available. Major corrections at proof stage, such as changes to figures, will be referred to the Editors for approval before they can be incorporated. Only minor changes, such as to style and consistency, should be made at proof stage. Changes that need to be made after proof stage will usually require a formal correction notice.

All queries at proof stage should be sent to: TJP@wiley.com

Are you on Twitter? Once your paper is online, why not share your achievement with your followers? Please tag The Journal (@jphysiol) in any tweets and we will share your accepted paper with our 30,000 followers!

Yours sincerely,

Laura Bennet
Senior Editor
The Journal of Physiology

P.S. - You can help your research get the attention it deserves! Check out Wiley's free Promotion Guide for best-practice recommendations for promoting your work at www.wileyauthors.com/eeo/guide. You can learn more about Wiley Editing Services which offers professional video, design, and writing services to create shareable video abstracts, infographics, conference posters, lay summaries, and research news stories for your research at www.wileyauthors.com/eeo/promotion.

• IMPORTANT NOTICE ABOUT OPEN ACCESS: To assist authors whose funding agencies mandate immediate public access to published research findings, The Journal of Physiology allows authors to pay an Open Access (OA) fee to have their papers made freely available immediately on publication.

The Corresponding Author will receive an email from Wiley with details on how to register or log in to Wiley Authors where you will be able to place an order.

You can check if your funder or institution has a Wiley Open Access Account here:
https://authors.wiley.com/author-resources/Journal-Authors/open-access/author-compliance-tool.html

REVIEWING EDITOR COMMENTS:

Thank you for the thorough revision of the manuscript. All concerns have been adequately addressed, and the work is now acceptable for publication. Congratulations!

REVIEWER COMMENTS:

REVIEWER 1:

The authors have satisfactorily addressed all of my concerns.

REVIEWER 2:

The manuscript has been revised in line with the reviewers' comments. In particular there is now a clearer consideration of the translational relevance of models (table 3). The contents of the review now better match the title and should provide a useful reference for those working in this field.

The authors have revised the manuscript to address my previous concerns.
